# Systematic miRNome profiling reveals differential microRNAs in transgenic maize metabolism

**DOI:** 10.1186/s12302-018-0168-7

**Published:** 2018-09-19

**Authors:** Sarah Zanon Agapito-Tenfen, Vinicius Vilperte, Terje Ingemar Traavik, Rubens Onofre Nodari

**Affiliations:** 1 GenØk–Centre for Biosafety, Forskningsparken i Breivika, Sykehusveien 23, 9294 Tromsø, Norway; 2 Departamento de Fitotecnia, Universidade Federal de Santa Catarina, Florianópolis, 88034000 Brazil; 3 Present Address: Institute for Plant Genetics, Faculty of Natural Sciences, Leibniz University of Hannover, 30419 Hannover, Germany

**Keywords:** miRNA, miRNome, GMOs, Biosafety, Risk assessment, *Zea mays*

## Abstract

**Background:**

While some genetically modified organisms (GMOs) are created to produce new double-stranded RNA molecules (dsRNA), in others, such molecules may occur as an unintended effect of the genetic engineering process. Furthermore, GMOs might produce naturally occurring dsRNA molecules in higher or lower quantities than its non-transgenic counterpart. This study is the first to use high-throughput technology to characterize the miRNome of commercialized GM maize events and to investigate potential alterations in miRNA regulatory networks.

**Results:**

Thirteen different conserved miRNAs were found to be dys-regulated in GM samples. The insecticide Bt GM variety had the most distinct miRNome. These miRNAs target a range of endogenous transcripts, such as transcription factors and nucleic acid binding domains, which play key molecular functions in basic genetic regulation. In addition, we have identified 20 potential novel miRNAs with target transcripts involved in lipid metabolism in maize. isomiRs were also found in 96 conserved miRNAs sequences, as well as potential transgenic miRNA sequences, which both can be a source of potential off-target effects in the plant genome. We have also provided information on technical limitations and when to carry on additional in vivo experimental testing.

**Conclusions:**

These findings do not reveal hazards per se but show that robust and reproducible miRNA profiling technique can strengthen the assessment of risk by detecting any new intended and unintended dsRNA molecules, regardless of the outcome, at any stage of GMO development.

**Electronic supplementary material:**

The online version of this article (10.1186/s12302-018-0168-7) contains supplementary material, which is available to authorized users.

## Background

Genetically modified (GM) crops intended for market release have been mostly created through in vitro DNA modification to encode a novel protein. However, a new generation of genetically modified organisms is being designed to change their RNA content in order to regulate gene expression [1]. The regulation of gene expression by RNA molecules, specifically double-stranded RNA (dsRNA), is now known to be possible through a process called RNA interference (RNAi). The biochemical pathway for this type of gene regulation can be generally understood as the disruption of the production of proteins when there are dsRNA molecules interacting with the DNA sequence of that gene. RNAi is an important biological pathway that is used by many different organisms to down-regulate the expression of endogenous or exogenous mRNA by the interaction of small interfering RNAs (siRNAs) and microRNAs (miRNAs) with catalytic RNA-induced silencing complex (RISC) to bind to homologous mRNA sequences and to cleave them [2]. miRNAs are known to control a wide range of biological processes in eukaryotic organisms, from embryogenesis [3], to developmental and cellular behaviour [4], and response to biotic [5] and abiotic stress [6].

miRNAs function as gene regulators and they have been shown to play important roles in multiple plant developmental and signalling pathways through small RNA biogenesis or related pathways [7]. Due to the environmental stability of dsRNAs, a property perhaps overlooked based on the relative instability of single-stranded species of RNA [8], GM crops can be engineered to produce dsRNAs that are harmful to insects and worms that feed on these plants [8–11]. On the other hand, studies have claimed that dsRNA molecules may rapidly degrade and dissipate in aquatic microcosms [12], and are biological inactive in agricultural soils after 2 days of exposure [13]. Nonetheless, the stability and transmissibility of dsRNAs suggest the potential for the existence of exposure routes that are relevant to environmental and human risk assessments of GM organisms [14]. In such cases, all new intended and unintended dsRNA molecules should be identified in the GM organism and products [15]. This should be obtained through a semi-targeted qualitative profiling of small RNA molecules, using next-generation sequencing in a comparative assessment of the GM and near-isogenic non-transgenic variety [14]. Since dsRNA produced by plants can target important mRNAs from pathogens feeding in the crop and cause lethality, there is a growing interest in using RNAi for pest and disease control, both as a traditionally applied insecticide/fungicide and within GM plants [16]. However, while some GMOs are intended to produce new regulatory-RNA molecules, these may also arise in other GMOs not intended to express them [14, 15, 17].

GM crops are regulated by international and national legislation and many countries adopt pre-market risk assessment to evaluate any risks that GM plants may pose to animal and human health and the environment [18, 19]. There are similarities and differences in the risks associated with RNAi-based GM plants relative to those posed by GM crops producing insecticide and herbicide-tolerant proteins [14]. As part of the pre-market risk assessment, many regulatory authorities evaluate all relevant scientific data on the molecular characterisation of the GM plant in question, such as the source and function of the donor DNA, the transformation method, the organization of the inserted DNA at the insertion site(s), and the expression and stability of the insert [18, 19].

Our previous paper documented risk assessment advice offered to government regulators during official risk evaluations of GM plants for use as human food or for release into the environment, how the regulator considered those risks, and what that experience teaches us about the GMO risk assessment framework [14]. We concluded that the process should include *inter alia* experimental procedures that would identify all new intended and unintended dsRNA molecules in the GM product and we have suggested improvements for such. The European Food and Safety Authority opinion is in agreement with our recommendations [1].

Since the evidence that exogenous plant miRNAs are present in the sera and tissues of various animals and that these exogenous plant miRNAs are primarily acquired orally through food intake [20], the safety of GMO-derived miRNAs has been debated [21–25]. Nonetheless, further studies have detected the presence of exogenous miRNAs in human samples from uncommon dietary sources (e.g. rodents), indicating that exogenous miRNAs originate from technical artefacts rather than dietary intake [26]. Hence, there are no conclusive evidences and consensus about this topic.

Only few efforts designed to investigate unique risk assessment issues related to GM crops or products containing dsRNA have been reported. The present study is the first that characterizes the miRNome of commercialized GM maize events to further investigate potential alterations in miRNA regulatory network. We were interested in testing miRNA content in two of the most commonly used transgenes in agriculture which currently do not produce intentional dsRNA; the insecticide Bt and the herbicide-tolerant Roundup Ready transgenes. The seed set of stacked (combination of transgenes by crossing or mating) and single GM maize events, as well as the non-transgenic near-isogenic counterpart, developed in the near-isogenic genetic background, enables the isolation of potential effects derived from transgenes independently.

Here, we show that the expression profile of several miRNAs is altered in GM varieties and that novel and potential transgenic miRNA sequences were detected. Although no unequivocal explanation was found for these phenomena, the interactions between miRNA, their targets and biochemical pathways are discussed. These analyses were carried out in order to create a documented record of the emergence of such alterations. The experimental approach of this study may also expand the knowledge about the potential risks of RNAi-based GM plants through the identification of potential changes that require further characterisation of products from cutting-edge technology.

## Results

### Deep sequencing of sRNA species in transgenic maize varieties

**Fig. 1:**
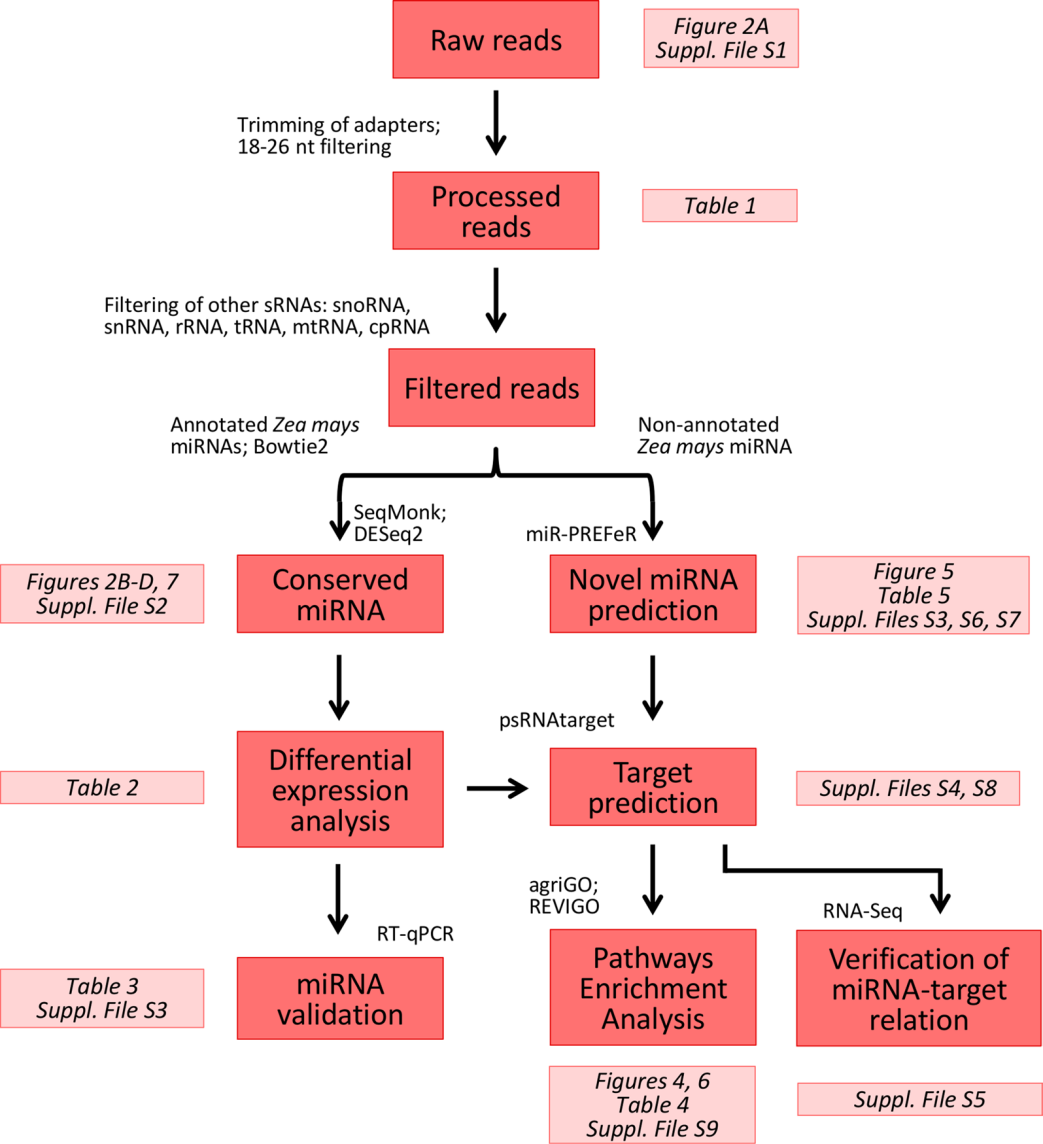
Schematic overview of the workflow implemented for the identification of differentially expressed regulatory miRNAs and their targets in GM and control maize samples

In order to identify conserved maize miRNAs in transgenic maize varieties, sRNA libraries were constructed from leaf material and sequenced using Illumina high-throughput technology. We have performed miRNA profiling in a unique set of stacked and single transgenic maize hybrid varieties, as well as the conventional counterpart, which were all developed under the same genetic background. Each sample consisted of a pool of 10 plants and three biological replicates were analysed, with a total of 30 plants assessed per variety. A flowchart of the miRNA analysis pipeline is available in Fig. 1. The full sequencing dataset is available through Sequence Read Archive (SRA) at NCBI under identification number SRP160077.

**Fig. 2:**
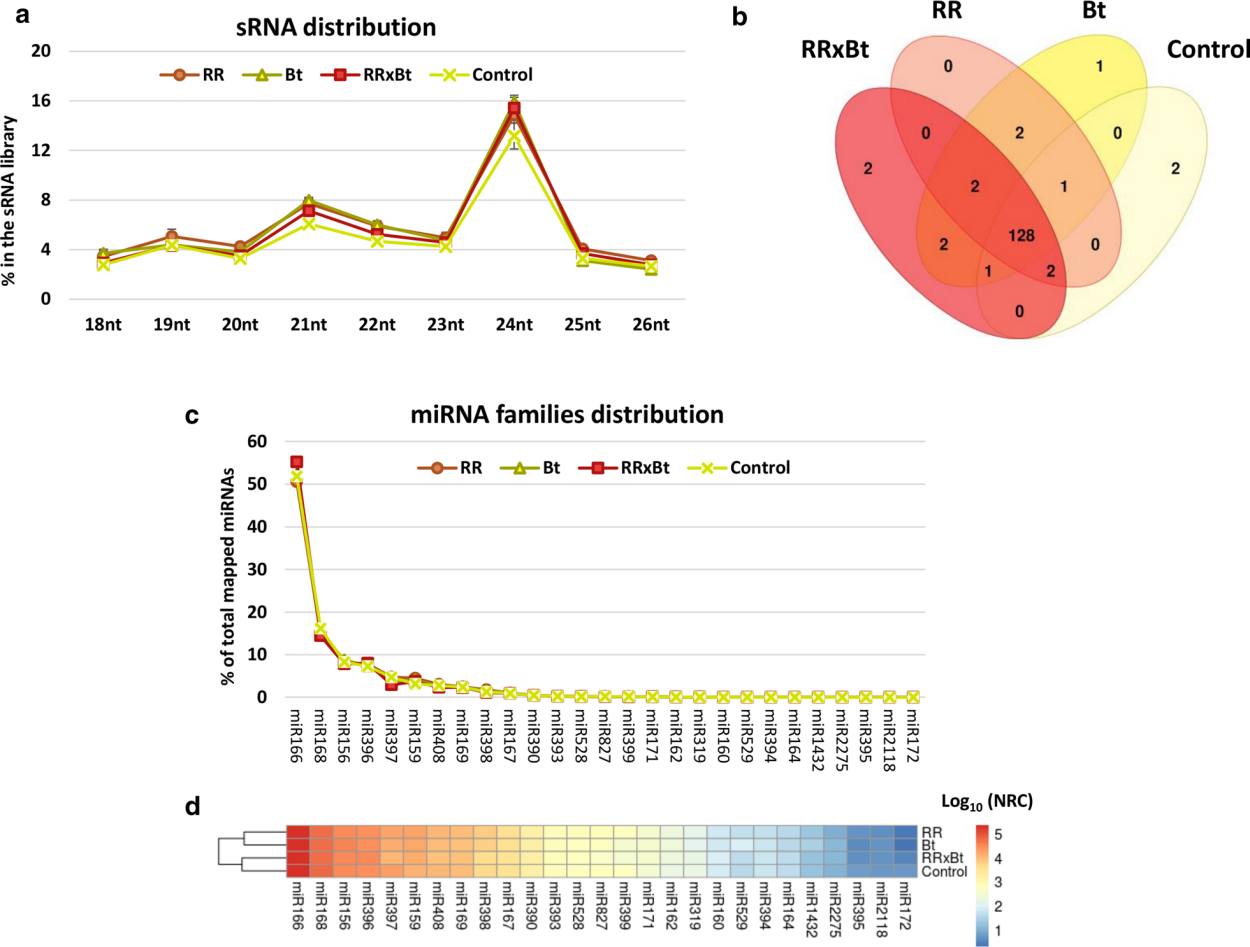
Alignment of sRNA reads with the Zea mays reference genome. **a** Size distribution (18–26 nt) of sRNA reads for each of the varieties analysed; **b** venn diagram showing the number of conserved miRNAs identified in each variety; **c** distribution of the identified conserved miRNAs along maize miRNA families for each of the analysed varieties; **d** heatmap of the normalized read count (NRC) for each of the conserved miRNA families in the analysed varieties. The NRC values were log10 transformed for better visualization

After removing low-quality sequences, the total library size ranged from 13,306,004 to 19,618,997 reads, with 44–54% belonging to the 18–26 nt range and thus used for further analyses. Reads with sizes < 18 and > 26 nt were excluded from the datasets. The read size distribution for all libraries is available in Additional file 1. The majority of the sRNAs with 18–26 nt belong to 24 nt size category, accounting for 13.2–15.9% of the total number of reads, followed by 21 nt (6.1–8.0%) and 22 nt (4.7–6.0%) (Fig. 2a). Similar distribution patterns were also observed in previous studies investigating plant miRNAs [27–30].

On average, approximately 22% of the reads matched to other types of non-coding sRNAs, such as rRNAs, tRNAs, snRNAs or snoRNA, and approximately 18% matched organellar RNA (chloroplastidial and mitochondrial) (Table 1). In addition, we have built a reference sequence database with the transgenic insert sequences of MON89034 and NK603 events, which was also used for mapping the reads. Transgenic sequences were detected in all GM varieties. The stacked variety showed a higher number of sequence reads (4908 reads) which was close to the sum of the reads in both single event varieties (476 read for the Bt variety and 4173 reads for the RR variety). Further investigation of the potential for those sequences to be an active miRNA was performed using miRPrefer prediction tool. The analysis did not yield significant results because the sequences did not fulfil the criteria for either precursor sequence size and/or maximum of 2 nt 3′ overhangs in mature-star duplex. No reads matching the transgenic sequences were found in control samples.

**Table 1:** Number of reads aligned to each sequence type for each of the four varieties used in the study

Sequence type	RR		Bt		RR×Bt		Control	
	Total reads	%	Total reads	%	Total reads	%	Total reads	%
snoRNA	1365	0.02	797	0.01	1454	0.02	1247	0.02
snRNA	7922	0.09	2989	0.04	3514	0.04	7937	0.10
tRNA	244,114	2.75	83,806	1.01	152,588	1.79	178,399	2.22
rRNA	1,710,288	19.30	2,003,376	24.09	1,568,675	18.38	1,528,139	19.01
cpRNA	1,724,918	19.47	1,100,942	13.24	1,531,116	17.94	1,526,939	19.00
mtRNA	53,568	0.60	48,856	0.59	69,729	0.82	64,551	0.80
Transgenic RNA	476	0.01	4,173	0.05	4908	0.06	–	–
miRNA	4,10,396	4.63	4,62,865	5.57	445,291	5.22	384,181	4.78
Other sRNAs	4,708,056	53.13	4,606,799	55.41	4,755,858	55.73	4,346,399	54.07

The B73 RefGen_v3 database was used as reference genome to search for conserved miRNA sequences. The overall distribution of miRNAs is represented by a Venn diagram in Fig. 2b. A total of 128 miRNAs were found to be shared among Bt, RR, RRxBt and control sample libraries, accounting for ~ 90% of the annotated miRNA detected in our bioinformatics analysis. Other miRNAs (10%) were assigned to be present in one variety only, these are: zma-miR2118c in control, zma-miR395f in RRxBt, zma-miR395g in Bt, and both zma-miR395d and zma-miR395 h in RR samples. Nonetheless, these exclusive miRNAs were only present in a few numbers of reads. Our sequencing effort presented a good miRNome coverage with a total of 404,728 to 428,487 number of reads per variety matching to 143 known miRNAs sequences from 27 miRNA families in maize (out of known 156 miRNA present on the reference genome) (Fig. 2c). The largest family was zma-MIR169 with 17 members, followed by zma-MIR171 (13 members) and zma-MIR156 and zma-MIR395 (11 members).

In relation to the abundance of each miRNA family, zma-MIR166 was the most present in all varieties (50.5 to 55.2% of the total miRNA content), followed by miR168 (14.5–16.2%) and miR156 (7.8–8.8%). Out of the top ten expressed miRNA families, our study shared 6 miRNA families matching the miRNA maize reference paper [30]. A heatmap with the normalized read count for all the identified miRNA families for each variety is shown in Fig. 2d. Although single event varieties group separately from stacked and control samples in the heatmap, there was no statistically distinct pattern of miRNA families’ abundance between varieties. The list of all identified maize miRNAs and the respective normalized read count for each variety is available in Additional file 2.

### Defining transgenic maize-specific microRNA signatures through multiple microRNA profiling comparisons

Pairwise differential expression analysis was performed based on the normalized read count using DESeq2 R package for each identified miRNA. To facilitate visualization, we have used the logarithm base 2 of the fold-change (Log2FC) as a measure of accumulation levels. Therefore, a gene up-regulated by a factor of 1.5 has a Log2FC of 0.58, a gene down-regulated by a factor of 1.5 has a Log2FC of − 0.58, and a gene expressed at a constant level (with a FC of 1) has a Log2FC equal to zero [31].

Thirteen known maize miRNAs were found to have statistically significant differences in abundance (*p*-*adjusted* < 0.05 was used as the only analytical threshold) (Table 2) between maize varieties. The results of the differentially abundant miRNAs were validated using SYBR reverse transcriptase quantitative PCR (RT-qPCR). The amplification of miRNA sequences is based on forward primers designed to match the full mature miRNA sequences, whereas the reverse primer is universal and targets poly-A tail added to miRNA sequences (Additional file 3).

**Table 2:** Log2Fold-Change, *p*- and *p*-*adjusted* values of the differentially expressed miRNAs

miRNA	Comparison	Log2Fold-Change	*p*-value	*p*-*adj* value
zma-miR162	Bt vs Control	0.5	0.0027	0.0275
zma-miR167c	Bt vs Control	0.6	0.0007	0.0266
zma-miR167e	Bt vs Control	0.5	0.0023	0.0275
zma-miR167j	Bt vs Control	0.6	0.0009	0.0266
zma-miR169f	Bt vs Control	− 0.8	0.0008	0.0266
zma-miR169 m	Bt vs Control	0.6	0.0025	0.0275
zma-miR399a	Bt vs Control	− 0.7	0.0014	0.0275
zma-miR399e	RR×Bt vs RR	− 0.9	0.0001	0.0044
zma-miR399 h	Bt vs Control	− 0.7	0.0023	0.0275
zma-miR399i	RR×Bt vs Control	− 0.6	0.0010	0.0311
	RR×Bt vs RR	− 1.0	0.0000	0.0007
zma-miR399j	RR×Bt vs Control	− 0.6	0.0005	0.0232
	RR×Bt vs RR	− 0.7	0.0009	0.0310
zma-miR529	Bt vs Control	0.7	0.0024	0.0275
zma-miR827	RR×Bt vs Control	− 0.5	0.0000	0.0006
	RR×Bt vs RR	− 0.5	0.0008	0.0310

Noteworthy, four miRNAs belonging to the same family could not be distinguished due to their identical mature miRNA sequence. We also observed that the amplification of the zma-MIR399eij showed a considerable difference when compared to the RNA-Seq results. This might be due to the homology of the designed primer to other members of the family, such as zma-MIR399c and zma-MIR399d, which might have hampered the results. In addition, zma-miR162 and zma-miR529 showed multiple melting curve peaks for the RT-qPCR analysis, thus precluding a reliable quantification of their expression. Overall, the comparative cycle threshold levels generated for most of the selected miRNAs were the same as those determined by Illumina sequencing, indicating that the sequencing data produced in this study were accurate. The comparison of Illumina and RT-qPCR quantification results is shown in Table 3.

**Table 3:** Comparative data of RT-qPCR and RNA-Seq for 7 differentially expressed conserved miRNAs

miRNA	Comp.	Samples	Ct Rep. 1	Ct Rep. 2	Ct Rep. 3	Read count	Log2FC RT-qPCR	Log2FC RNA-Seq
zma-MIR167c	Bt vs Control	Bt S1	26.59	26.56	26.68	103	0.58	0.56
		Bt S2	26.67	26.49	26.62	304		
		Bt S3	26.66	26.37	26.38	230		
		Conv S1	27.33	27.31	27.58	199		
		Conv S2	27.53	27.85	27.69	132		
		Conv S3	27.39	26.97	26.79	170		
zma-MIR167ej	Bt vs Control	Bt S1	26.22	26.17	26.11	325	0.11	0.55
		Bt S2	25.99	25.95	25.93	515		
		Bt S3	26.11	26.17	26.10	428		
		Conv S1	26.46	26.53	26.54	336		
		Conv S2	26.55	26.65	26.49	293		
		Conv S3	26.24	26.31	26.11	380		
zma-MIR169f	Bt vs Control	Bt S1	26.81	26.34	26.27	108	− 1.05	− 0.78
		Bt S2	26.35	26.87	27.07	118		
		Bt S3	26.43	26.13	26.73	170		
		Conv S1	25.11	24.88	25.09	77		
		Conv S2	26.61	25.63	25.76	176		
		Conv S3	26.80	26.84	26.62	115		
zma-MIR169 m	Bt vs Control	Bt S1	28.12	27.99	28.14	561	0.42	0.62
		Bt S2	27.36	27.42	27.53	852		
		Bt S3	27.56	27.81	27.14	642		
		Conv S1	28.95	28.27	28.51	447		
		Conv S2	28.33	28.41	27.90	617		
		Conv S3	28.40	28.27	27.89	433		
zma-MIR399ah	Bt vs Control	Bt S1	29.68	29.44	29.75	26	− 0.29	− 0.70
		Bt S2	29.43	29.36	29.14	48		
		Bt S3	29.22	29.34	29.12	34		
		Conv S1	29.41	29.09	28.90	55		
		Conv S2	29.69	29.19	29.62	40		
		Conv S3	28.98	29.99	28.99	44		
zma-MIR399eij	RR×Bt vs Control	RR×Bt S1	22.15	22.09	22.12	63	− 1.51	− 0.64
		RR×Bt S2	22.27	22.35	22.31	91		
		RR×Bt S3	22.47	22.36	22.29	107		
		Conv S1	20.61	20.66	20.72	116		
		Conv S2	21.09	21.08	21.07	107		
		Conv S3	20.71	20.71	20.82	165		
zma-MIR399eij	RR×Bt vs RR	RR×Bt S1	22.15	22.09	22.12	63	0.00	− 0.86
		RR×Bt S2	22.27	22.35	22.31	91		
		RR×Bt S3	22.47	22.36	22.29	107		
		RR S1	23.11	23.09	23.14	146		
		RR S2	22.10	22.07	22.16	203		
		RR S3	23.39	23.46	23.41	150		
zma-MIR827	RR×Bt vs Control	RR×Bt S1	27.27	26.97	26.99	479	− 0.58	− 0.54
		RR×Bt S2	27.33	27.35	27.52	552		
		RR×Bt S3	27.37	27.35	27.25	554		
		Conv S1	26.92	26.84	26.63	683		
		Conv S2	26.53	26.87	27.18	602		
		Conv S3	26.98	26.53	26.67	675		
zma-MIR827	RR×Bt vs RR	RR×Bt S1	27.27	26.97	26.99	479	− 1.00	− 0.50
		RR×Bt S2	27.33	27.35	27.52	552		
		RR×Bt S3	27.37	27.35	27.25	554		
		RR S1	26.69	26.64	26.70	656		
		RR S2	26.80	26.81	27.00	718		
		RR S3	26.74	26.59	26.54	751		

zma-miR162 and zma-miR529 are not included in the table due to multiple melting curve peaks on the RT-qPCR. *Comp.* comparison, *Ct* cycle threshold, *Rep.* replicate, *FC* fold change, Control conventional non-transgenic sample, *Bt* MON89034 transgenic event sample, *RR* NK603 transgenic event sample, *RR×Bt* stacked transgenic event

One of the challenges in elucidating the biological functions of miRNAs is to identify their regulatory targets. We have predicted targets for the 13 differentially regulated miRNAs using psRNAtarget and found 116 potential endogenous transcript targets. These included splicing variants and gene expression inhibition by either mRNA cleavage or translational inhibition. The list of all miRNA targets is available in Additional file 4. The miRNAs with the largest number of mRNA targets were zma-miR529-5p (47) and zma-miR169f-5p (25).

As expected, many of the putative targets of maize conserved miRNA identified were transcriptions factors (TFs) mRNAs, which is considered a general trend for plant miRNAs [7]. Of the 116 predicted targets, 24 are members of transcription factor gene families (i.e. member of the nuclear transcription factor Y subunit A-3 family, squamosa promoter-binding protein-like (SBP domain) transcription factor family, putative GATA transcription factor family and the Ocs element-binding factor 1-putative bZIP transcription factor superfamily) involved in many central developmental and physiological processes, including morphogenesis, abiotic and biotic stress responses.

**Fig. 3:**
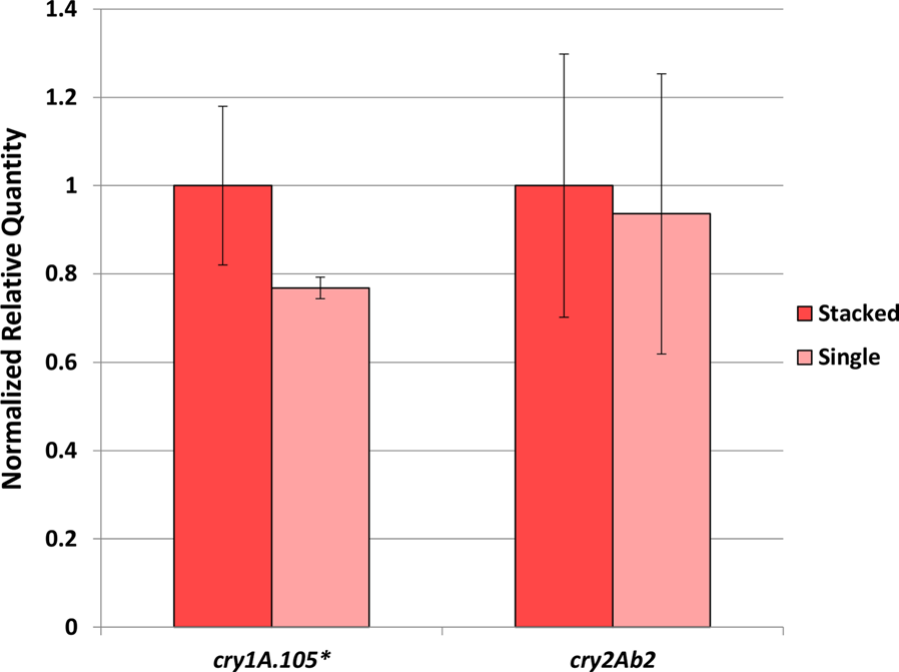
Transgene transcripts normalized relative expression levels measured by delta-delta Cq method and Pffafl correction equation. The cry1A.105 and cry2Ab2 transgenes were quantified in single versus stacked transgenic maize events grown under controlled conditions at V3 stage. Samples are means of three pools, each derived from ten different plants. ‘Bt’ samples are from MON-89Ø34-3 event, and ‘RR×Bt’ samples are transgenic maize seedlings from MON-89Ø34-3 × MON-ØØ6Ø3-6 event. Bars indicate standard deviation and statistically significant values (*p* < 0.05) are represented by ‘*’

The miRNome profile of Bt-expressing transgenic plants was the most distinct one showing nine out of 13 dys-regulated miRNAs from five miRNA families (zma-MIR162, zma-MIR167, zma-MIR169, zma-MIR399 and zma-MIRNA529). We have further focused our downstream analysis of the Bt miRNome to explore possible factors influencing miRNA accumulation in this variety. We were able to verify a statistically significant increase of cry1A.105 protein transcript accumulation in Bt versus stacked event through RT-qPCR (Fig. 3). Although these results do not directly correlate to the most altered miRNA profile in the Bt variety, the expression and accumulation of CRY proteins suggest alterations in the miRNome of both single and stacked varieties, which showed the two most distinct miRNA profiles. However, because CRY protein in a non-native maize protein, it is not possible to verify annotated pathway networks that integrate CRY.

**Fig. 4:**
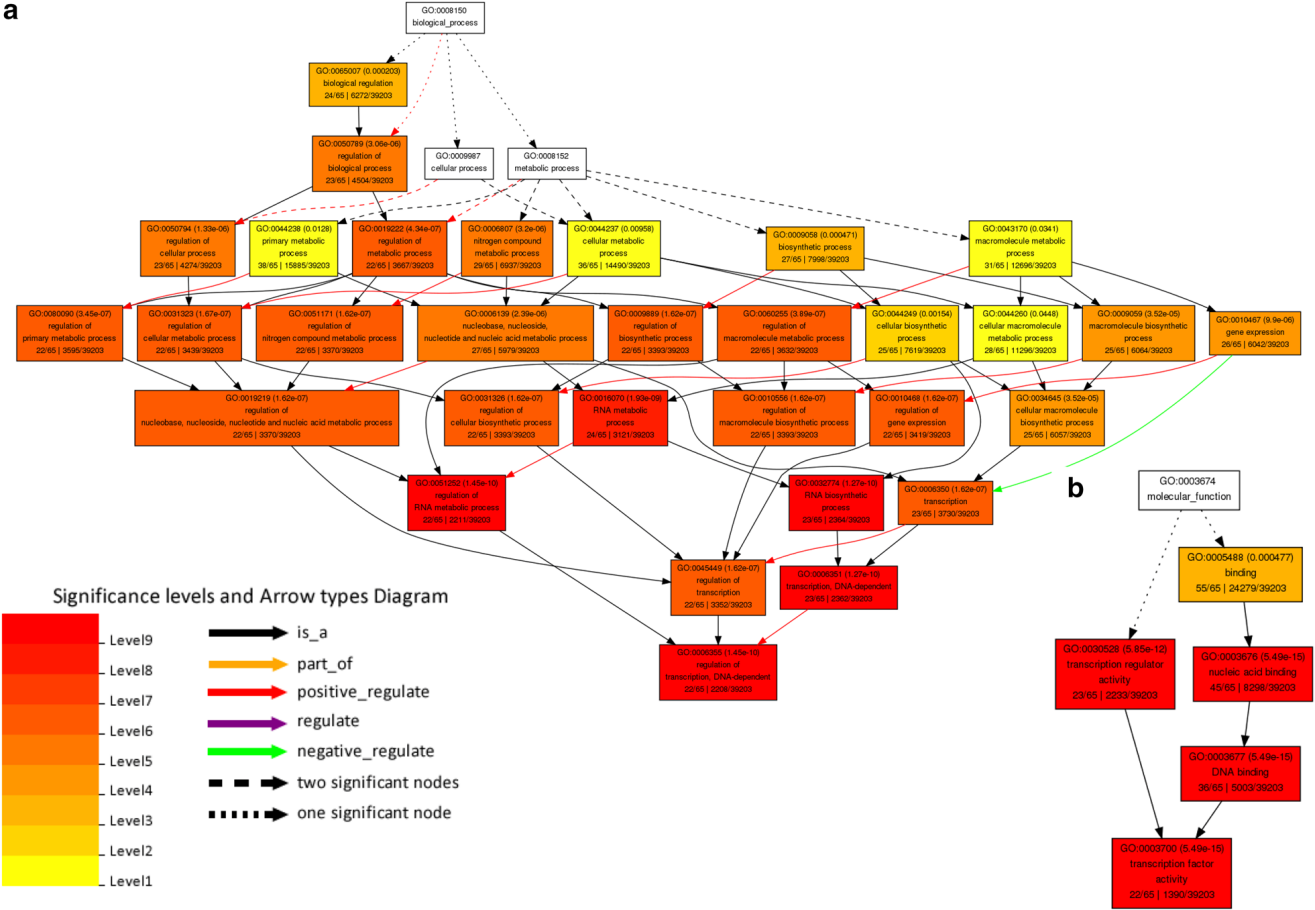
Significantly enriched pathways for the differentially expressed conserved miRNA targets in Bt-expressing plants. **a** Differentially regulated pathways for biological processes; and **b** for molecular function (FDR < 0.05) analysed by agriGO online tool

In order to gain insight into what metabolic pathways would be affected by the altered miRNAs in the Bt variety, we submitted transcript targets of these differentially regulated miRNAs to a singular pathways enrichment analysis using agriGO toolkit. We observed that these transcripts were mainly assigned to RNA biosynthetic process (GO:0032774), regulation of DNA-dependent transcription (GO:0006355) and regulation of RNA metabolic process (GO:0051252) (Fig. 4a). Examples of mRNA targets associated with these pathways are dicer-like 1 protein family, Pumilio-family RNA binding domain, Histone H4.3, Nucleic acid binding protein NABP, Nuclear transcription factor Y subunit A-3 isoform 2, squamosa promoter-binding protein-like (SBP domain) transcription factor family protein, putative GATA transcription factor family protein, among others. These proteins shared common molecular functions of transcription regulator activity (GO:0030528, GO:0003700) and nucleic acid binding (GO:0003676, GO:0003677) (Fig. 4b and Table 4).

**Table 4:** Significant biological processes and molecular function categories targeted by differentially expressed microRNAs (miRNAs) in insecticide expressing Bt GM maize plants

GO ID	Term description	Number of transcripts	FDR
Biological processes			
GO:0032774	RNA biosynthetic process	22	1.80E−11
GO:0006351	Transcription, DNA-dependent	22	1.80E−11
GO:0051252	Regulation of RNA metabolic process	21	2.40E−11
GO:0006355	Regulation of transcription, DNA-dependent	21	2.40E−11
GO:0016070	RNA metabolic process	23	2.10E−10
GO:0031326	Regulation of cellular biosynthetic process	21	2.00E−08
GO:0045449	Regulation of transcription	21	2.00E−08
GO:0019219	Regulation of nucleobase metabolic process	21	2.00E−08
GO:0009889	Regulation of biosynthetic process	21	2.00E−08
GO:0051171	Regulation of nitrogen compound metabolic process	21	2.00E−08
GO:0010556	Regulation of macromolecule biosynthetic process	21	2.00E−08
GO:0006350	Transcription	22	2.00E−08
GO:0010468	Regulation of gene expression	21	2.10E−08
GO:0031323	Regulation of cellular metabolic process	21	2.20E−08
GO:0080090	Regulation of primary metabolic process	21	4.40E−08
GO:0060255	Regulation of macromolecule metabolic process	21	4.90E−08
GO:0019222	Regulation of metabolic process	21	5.40E−08
GO:0050794	Regulation of cellular process	22	1.40E−07
GO:0050789	Regulation of biological process	22	3.40E−07
GO:0010467	Gene expression	25	7.80E−07
GO:0034645	Cellular macromolecule biosynthetic process	24	3.10E−06
GO:0009059	Macromolecule biosynthetic process	24	3.10E−06
GO:0006807	Nitrogen compound metabolic process	25	8.80E−06
GO:0006139	Nucleobase	23	8.80E−06
GO:0065007	Biological regulation	23	1.90E−05
GO:0009058	Biosynthetic process	26	3.20E−05
GO:0044249	Cellular biosynthetic process	24	0.00013
GO:0043170	Macromolecule metabolic process	30	0.0022
GO:0044260	Cellular macromolecule metabolic process	27	0.0037
GO:0044238	Primary metabolic process	34	0.0046
GO:0044237	Cellular metabolic process	31	0.0084
Molecular functions			
GO:0003677	DNA binding	35	3.00E−16
GO:0003700	Transcription factor activity	21	2.40E−15
GO:0003676	Nucleic acid binding	41	4.00E−15
GO:0030528	Transcription regulator activity	22	1.10E−12
GO:0005488	Binding	49	0.00041

FDR values indicate the relevance of the function, with lower values suggesting higher relevance

Stacked transgenic event plants had the second most distinct miRNome with four miRNAs (zma-miR399e, zma-miR399i, zma-miR399j and zma-miR827) being differentially accumulated when compared to control or to single event expressing CP4-EPSPS. The three zma-MIR399 species found to be less accumulated in stacked event target the same mRNA assigned to contain an HSF-type DNA-binding domain. On the other hand, zma-miR827 targets 13 different gene transcripts, among them transcripts containing domains of WD40-like Beta Propeller Repeat, BTB/POZ domain, SPX-Major Facilitator Superfamily domain and Myb/SANT-like DNA-binding domain (Additional file 4).

We have performed independent gene target profiling using RNA-Seq analysis to evaluate the expression of predicted target transcripts in all varieties and the non-transgenic control (Additional file 5). The RNA-Seq differential analyses showed 77, out of 116, putative miRNA-predicted target relations. Although there is no established threshold for the miRNA-target ratio, computational prediction and experimental validation have demonstrated negative correlations among miRNA-target pairs due to mRNA degradation which were also observed in this study [32].

Overall, the results showed that 13 miRNAs were differentially modulated in different pairwise comparisons. The results also suggest that CRY proteins can be considered a major factor influencing the miRNome of both Bt and stacked events in an additive manner. The altered miRNAs can target a range of endogenous transcripts, mostly transcription factors and nucleic acid binding domains that are key molecular functions for basic genetic regulation and some have been confirmed by independent RNA-Seq analysis.

### Prediction of novel miRNA candidates and their potential targets

In order to identify novel miRNAs sequences in our samples, we have used the miR-PREFeR pipeline, together with MiPred online tool, to predict their pre-miRNA structure. A total of 20 putative novel miRNAs have been identified in one or more varieties (Table 5).

**Table 5:** Location in the genome, miRNA sequence and number of mapped reads for the 20 putative novel miRNAs identified in maize varieties

miRNA ID	Location on genome	miRNA sequence	Read count from miR-PREFeR				Ct from RT-qPCR			
			RR	Bt	RR×Bt	Control	RR	Bt	RR×Bt	Control
zma-miRX01	chr01|95866804: 95866892	AUGGAGUGGAUUGAGGGGGCU	36	51	43	50	–	–	–	–
zma-miRX02	chr01|224709986:224710116	AUCCGGUACAAACGAACAAGGCCU	168	179	162	130	–	–	–	–
zma-miRX03	chr10|5127325: 5127461	AGAGUGGACAGUUGACGCCGGCCC	0	0	0	152	22.0	21.4	21.6	21.9
zma-miRX04	chr10|12355028: 12355155	AAUACAUGUGGAUUGAGCUCAAUA	42	57	43	45	–	–	–	–
zma-miRX05	chr10|71614450: 71614580	AUCCGACAGAAACGAACAAGGCCU	615	0	0	607	24.8	24.4	24.7	25.2
zma-miRX06	chr10|120859137:120859262	UAUUCGAGAACGGAUGUAGUACAU	546	672	655	525	31.1	33.9	31.1	33.9
zma-miRX07	chr10|145006838:145006964	AUUAGGGUAGAACCGAACAAGCCU	50	55	56	65	–	–	–	–
zma-miRX08	chr02|30270820: 30270916	AAUACAUGUGGAUUGAGCUCAAUA	42	57	43	45	–	–	–	–
zma-miRX09	chr02|144495862:144495992	AUCCGACGCAAACGAACAAGGCCU	78	109	102	68	–	–	–	–
zma-miRX10	chr02|203809131:203809256	AGGGUAUUGAUAGGACUAUAAUCC	352	351	323	373	28.6	28.1	29.5	28.9
zma-miRX11	chr02|229799695:229799756	GGGGAUGUAGUUCAGAUGGUAGAA	5893	0	0	0	29.7	30.1	29.5	29.7
zma-miRX12	chr03|162601521:162601594	UGUUUGGGAUUAUAAUCUGCC	47	71	48	56	–	–	–	–
zma-miRX13	chr03|178085743:178085863	AAAUACUGUAGAAGCCGCAGCCGC	0	2937	0	0	–	17.2	–	–
zma-miRX14	chr04|100693925:100694040	AGAGUGGACAGUUGACGCCGGCCC	0	0	0	152	–	–	–	–
zma-miRX15	chr05|144731639:144731774	ACGAGAGAGGACGUCAGGGGACGA	11	29	27	27	–	–	–	–
zma-MIR16	chr05|174841383:174841471 chr05|174842606:174842694 chr05|174912223:174912311 chr05|174938499:174938587	CUGAGCAAAAAAACACGACUAAG	21	26	23	16	–	–	–	–
zma-MIR17	chr07|104640325:104640456	AUUCCGGAACAAACGAACACACCC	18	17	18	28	–	–	–	–
zma-MIR18	chr07|123915132:123915213	UUUGAGAUUCGUAGCUUUUAC	77	79	95	77	–	–	–	–
zma-MIR19	chr08|61110522: 61110652	AUCUGACACAAACGAACAAGGCCU	82	92	94	77	–	–	–	–
zma-MIR20	chr08|171925082:171925167	ACGGAUCAAAUCUAUGGUGAGAUU	53	78	59	58	35.1	36.2	34.7	35.1

miRNA zma-miRX03 and zma-miRX14, as well as zma-miRX04 and zma-miRX08, show identical mature sequences, but their pre-miRNAs have different sequences. “–” means either no available primer for that miRNA sequence or unspecific melting curve

**Fig. 5:**
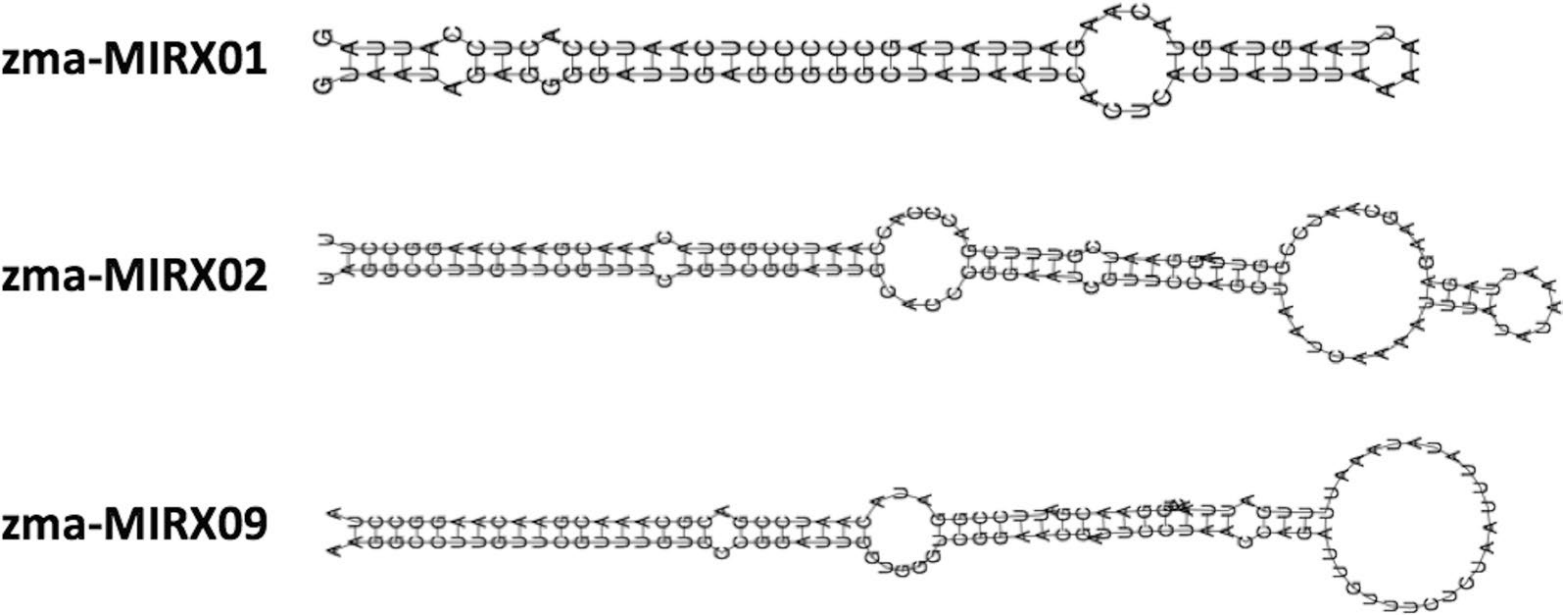
Prediction of novel maize miRNAs. Examples of the structures of three predicted novel maize miRNAs. The prediction was performed using the PREFeR pipeline, which uses RNAfold (from the ViennaRNA package) algorithm to calculate and draw the pre-miRNA hairpin structures

Detailed information of novel miRNAs is available in Additional file 6. The novel miRNAs sequences were temporarily named as ‘zma-MIR-number’ followed by an ‘X’ to differentiate them from the conserved maize miRNAs (e.g. zma-MIRX01). The predicted structure of all 20 novel miRNAs is available in the Additional file 7, and three of these novel miRNAs are shown in Fig. 5. Out of the 20 novel miRNAs, two were exclusively detected in control samples (zma-MIRX03 and zma-MIRX14), one in the RR samples (zma-MIRX11) and one in the Bt samples (zma-MIRX13).

Confirmatory analysis of novel miRNA was performed by RT-qPCR for those which was possible to design primers (Additional file 3). We were able to obtain single amplicons by melting curve analysis for only 7 miRNA primers. All these 7 miRNAs (zma-miRX03, zma-miRX05, zma-miRX06, zma-miRX10, zma-miRX11, zma-miRX13 and zma-miRX20) have provided positive amplifications via RT-qPCR (Table 5). Surprisingly, amplifications were also obtained in samples that showed no miRNA reads by miRNome sequencing. Spurious amplifications might be explained by the amplification of similar or identical miRNA mature sequences originated from different pri-miRNA loci. Poor sequencing of the sample and/or limitations of the bioinformatics analysis is also a possibility. In fact, in spite of the availability of a few amplification-based platforms for miRNA detection, the common technical challenges still exist, namely sensitivity, specificity especially for one base variation, selectivity between precursor and mature miRNAs [33].

**Fig. 6:**
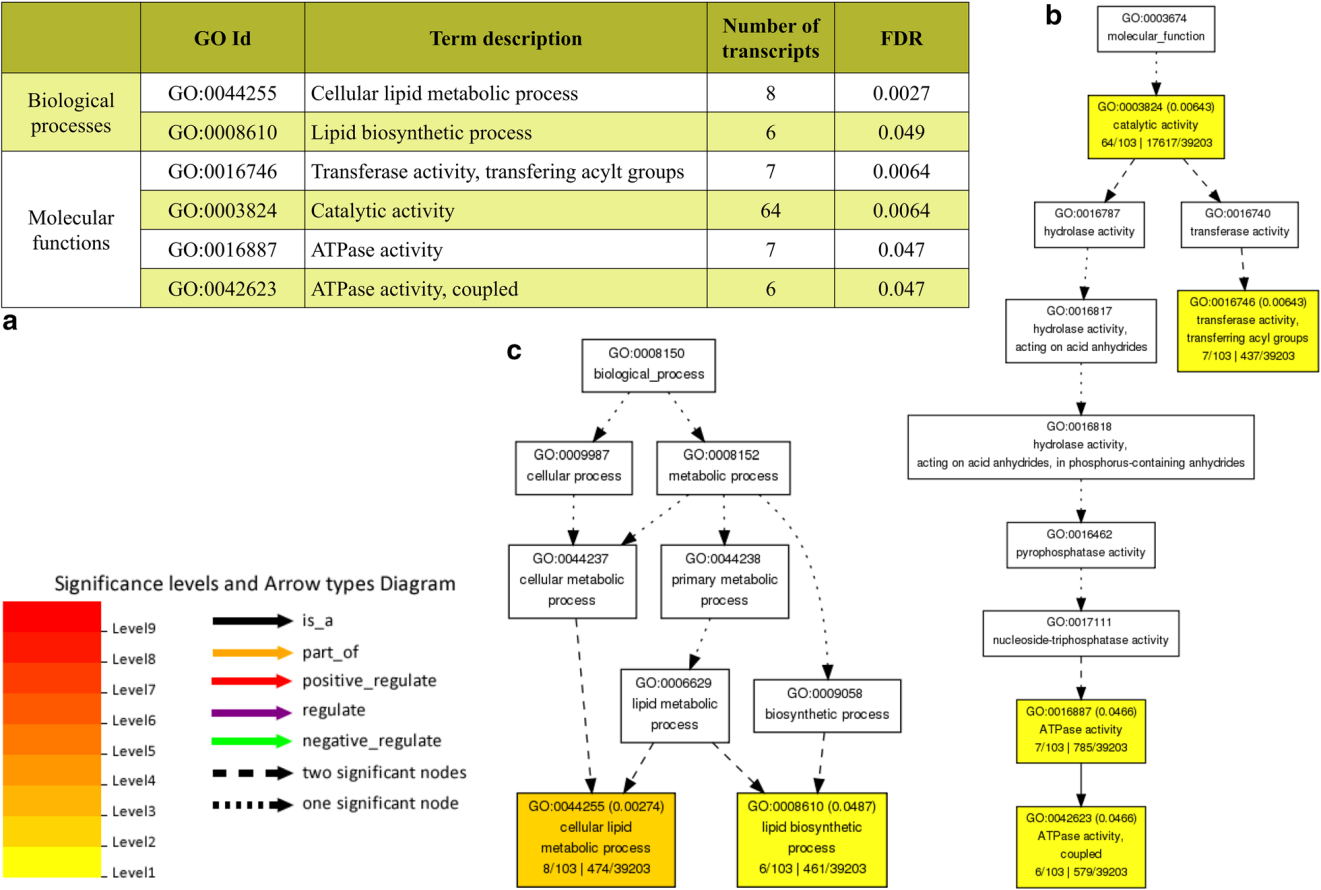
Pathways enrichment analysis of the miRNA targets from the 15 novel miRNAs predicted in this study. **a** List of the differentially regulated (FDR < 0.05) GO terms; **b** differentially regulated pathways for biological processes; and **c** differentially regulated pathways for molecular function (FDR < 0.05) analysed by agriGO online tool

The prediction of the novel miRNA targets showed 420 potential endogenous transcript targets, including splicing variants and gene expression inhibition by either mRNA cleavage or translational inhibition. The list of all novel miRNA targets is available in Additional file 8. Transcripts targets of miRNAs shared by all samples were submitted to singular pathways enrichment analysis using agriGO toolkit. Only two GO terms (*p* value < 0.05) were significantly enriched for biological processes in the miRNA targets of the predicted novel miRNAs, cellular lipid metabolic process (GO:0044255) and lipid biosynthetic process (GO:0008610). Four major molecular functions had significant GO term hits and most transcripts were assigned to catalytic activity (GO:0003824) (Fig. 6 and Additional file 9). The two novel miRNAs identified in control samples only (zma-MIRX03 and zma-MIRX14) had identical targets and those were mainly identified as Det1 complexing ubiquitin ligase (DDA1), putative calcium-dependent lipid-binding (CaLB domain) family protein and phosphate:H+ symporter. Exclusive targets for novel miRNAs in RR samples were found related to cytochrome b5 and leucine-rich repeat (LRR) protein families. Exclusive target transcripts in Bt samples were assigned to several gene families, such as caleosin-related protein, RHO protein GDP dissociation inhibitor, putative DUF231 domain containing family protein, lipid-binding START domain of mammalian STARD2, -7, thylakoid soluble phosphoprotein (TSP9) superfamily, among others (Additional file 8).

### Identification of miRNA variants or “isomiRs”

**Fig. 7:**
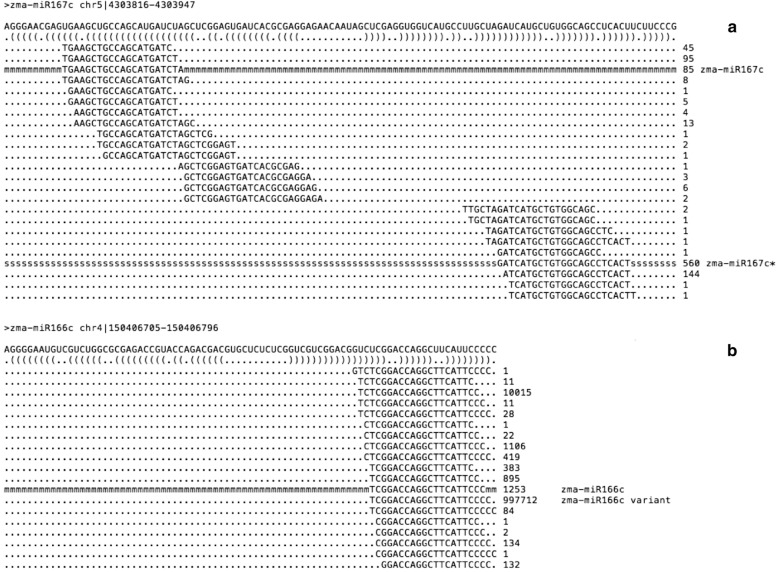
Distribution of sRNAs along conserved miRNA precursors. **a** Example where the corresponding miRNA* is more abundant than the annotated miRNA; **b** example where the most abundant sRNA is not the annotated miRNA or the miRNA*, but one of its variants

The miR-PREFeR pipeline was used to analyse possible variant sequences arising from the miRNA precursor. We considered as variants, or isomiRs, any sequence that mapped to the miRNA precursor and was neither identified as being a mature nor a star sequence by the pipeline. Out of 156 annotated miRNAs in maize genome (B73, RefGen_v3, release 25), the algorithm was able to predict 96 miRNA sequences, with all of them showing at least one variant sequence. In Fig. 7, we present two cases of such miRNA variants. In the first example, the corresponding miRNA* is more abundant than the annotated miRNA (Fig. 7a). In the second example, the most abundant sRNA is neither the annotated miRNA nor the miRNA*, but one of its variants (Fig. 7b).

## Discussion

In this report, we aimed at identifying which correlation of the differentially expressed miRNAs is more likely to play roles in transgenic maize through miRNA-gene interaction networks compared to non-transgenic near-isogenic control. Unless the GM plant is intended to produce new dsRNA molecules, the RNA itself is rarely formally considered in a risk assessment [14]. This is surprising since many GMO risk assessment guidance draw special attention to the characterization of novel RNAs, frequently mentioning the need to provide information on any expressed substances in the recombinant-DNA plant, such as: “any gene product(s) (e.g. a protein or an untranslated RNA)” (paragraph 32 of [34]); “demonstrate whether the inserted/modified sequence results in intended changes at the protein, RNA and/or metabolite levels” (page 10 of [35]); “detailed description of the expression of the gene product inserted in the host organism” (item 10, Annex II [36]). Although the GM varieties studied here were not tested for their dsRNA content during approval process because of assumptions made either explicitly or implicitly in the context of risk assessments, our detailed analysis of MON89034, NK603 and stacked MON89034xNK603 suggests that their miRNA profiles are not equivalent to the near-isogenic non-transgenic control samples.

We have developed a study design helping to fill the vacuum for the risk assessment of dsRNAs unique to, or present at specific concentrations in, GMOs. We have also identified areas of research that need technical improvements as well as areas of current uncertainty related to specific aspects of miRNA biology that require further investigation. Our study may be useful in the first step of a sequential approach to assess the potential for adverse effects arising from dsRNA-initiated modifications to organisms before bioinformatics and exposure analysis are performed. We have sampled GM plants grown under strictly controlled conditions and we have compared their miRNA profile to unmodified near-isogenic control lines that have the same genetic background as the GM varieties so to isolate the differences in miRNA content only. Profiling approaches have been long recommended by EFSA for the allergenicity assessment of the whole GM plant [37]. In addition, a recent report from EFSA’s workshop on ‘Risk assessment considerations for RNAi-based GM plants’ also highlighted the need for more research on metabolic profiling of such plants to support risk assessment through the use of various ‘omics’ techniques [1]. Although comparative miRNA profiling has not yet been performed in commercialized GM crops, other layers of genetic information, such as the proteome and metabolome, have been widely applied and shown to be a useful source of information for the risk assessment. Many studies have investigated the proteomes, transcriptomes and metabolomes in grains and leaves of insect-resistant and herbicide-resistant maize and suggested metabolic alterations due to the genetic transformation process [38–42]. Due to a more recent wave of omics datasets that have started to be used in some risk assessment areas and have been accepted as a powerful tool to substitute or complement current studies, EFSA has also hosted a dedicated colloquium early this year to move towards the implementation of omics techniques to GMO risk assessment in Europe [43].

Remarkable findings of ingested plant miRNA in animal liver and blood have been demonstrated by a Chinese study and this directed the attention of GMO regulators to potential cross-kingdom regulation between GM plants and higher organisms. Plant microRNAs have been detected in human blood, demonstrating that dsRNAs can survive digestion and be taken up from the gastrointestinal tract [20]. The plant-derived dsRNA molecules silenced an endogenous gene in human tissue culture cells, and in mouse liver, small intestine and lung [20]. Other studies have also shown that regulatory RNAs from plants can be detected in animals, including humans [24, 44–46], and it was found that some dsRNAs from plants were more frequently present in animal tissues and blood than predicted from their level of expression in plants. Other plant-derived dsRNAs (i.e. rice, corn, barley, tomato, soybean, wheat, cabbage, grapes and carrot) have later on been detected in humans [47]. Although the functionality of such exogenous RNAs in terms of mediating gene expression in animals has been controversial [48, 49], there is enough evidence that such RNAs are capable of translocating from the host to its interacting organism, and vice versa [50]. Overall, there is an enormous lack of knowledge about the biological significance of the uptake of plant-derived miRNAs and, therefore, further investigations are required [49].

In the current study, we performed miRNA expression profiles, as well as miRNA target prediction analysis to identify a subset of miRNAs that could be correlated with transgenic maize varieties that expressed insecticidal CRY protein and the EPSPS protein that confers herbicide tolerance. The transgenic plants encoding two CRY toxins, event MON89034, revealed the most distinct miRNome profile followed by the profile of the stacked transgenic event. Yet, the distinct miRNome is not correlated to a higher expression of CRY transcripts in Bt single event variety. Our independent RT-qPCR analysis revealed similar expression of Cry2Ab2 and a significantly decreased expression of Cry1A.105 in single compared to the stacked variety. In fact, quantification of transgenic transcripts and its correlation to protein accumulation has frequently yielded inconsistent results [51].

We have then examined the relationship of altered miRNAs to pathways that are perturbed by the corresponding miRNA gene targets in Bt samples. Nine miRNAs from five miRNA gene families were exclusively up-regulated in Bt plants (except for zma-MIR399 which was down-regulated in Bt samples). Metabolic pathway enrichment analysis revealed that relevant gene targets were related to RNA metabolic processes and regulation of DNA-dependent transcription. These observations suggest built-in redundancy of overlapping gene targets regulated by different miRNAs targeting the same set of pathways. The potential for functional redundancy among plant miRNAs has been previously studied by inactivation of the entire MIR164 family in Arabidopsis [52]. It was found that mir164abc triple-mutant plants showed severe defects in flower development and phyllotaxis, and these defects were not observed in plants in which only individual MIR164 genes are disrupted. Thus, there were indications that miR164 miRNAs are controlling shoot development in a redundant manner, while the degree to which individual miR164 miRNAs contribute to the regulation of different developmental processes varies [52]. Therefore, differences in the expression patterns of the individual miRNA genes imply that redundancy among them is not complete and that these miRNAs show functional specialization, which may pose extra challenges for any single miRNA to be used as a target for further studies on its potential biosafety implications.

Another altered metabolism in Bt samples was related to primary metabolic process (GO:0080090 and GO:0044238) and nitrogen compound metabolism (GO:0051171 and GO:0006807). Primary metabolism in plants is directly involved in growth, development, and reproduction, involving basic processes such as carbohydrate, lipid and protein metabolism. Our previous proteomic investigation using the same GM varieties also showed differential modulation of enzymes that catalyse chemical reactions involved in carbohydrate metabolism [40]. Overall, other comparative proteomic studies of transgenic versus non-transgenic crops corroborate these results. In fact, the energetic metabolism, including the carbohydrate metabolism, has been the most frequently observed metabolism category within comparative proteomic analysis of transgenic versus non-transgenic crops (see compilation at Table 3 from [41, 53]).

Two miRNAs families had several miRNAs being altered, zma-miRNA167 in Bt plants and zma-miRNA399 mostly in stacked plants. zma-miR167 is known to target auxin response factor (ARF) genes [54]. The phytohormone auxin influences many aspects of plant development and the identity of several miRNA targets suggests roles for miRNAs in auxin signalling [55]. ARFs are a plant-specific family of DNA binding proteins that bind to auxin-responsive promoter elements (AuxREs), which are found in early auxin response genes, including Auxin/Indole-3-Acetic Acid (Aux/IAA), small auxin-up RNA (SAUR), and auxin-inducible GH3 genes, and can either enhance or repress transcription [56]. The role of auxin dys-regulation in Bt plants is yet unknown but might be related to the CRY protein pathway as part of the plant defence system against pathogens [57]. zma-MIR399 was less abundant in stacked plants and showed a target transcript that contains a plant calmodulin-binding domain. Calmodulin-binding proteins are thought to be involved in diverse pathways that are dependent on Ca^2+^ signalling, such as plant development and adaptation to environmental stimuli [58]. In addition, also less abundant, zma-MIR827 targets another class of transcription factors, such as the bZIP transcription factor superfamily, the Ocs element-binding factor 1 and the Rossmann-fold superfamily protein. In plants, basic region/leucine zipper motif (bZIP) transcription factors regulate several processes including pathogen defence and stress signalling [59].

Off-target effects of ncRNAs producing plants have been observed. Tomato plants expressing short-hairpin RNAs targeting a replicase protein of Tomato leaf curl New Delhi virus (ToLCNDV) showed phenotypic abnormalities, such as needle-shaped leaves, reduced stature, and decreased lateral rooting, among others [60]. Similarly, soybean plants containing RNAi construct aimed to silence soybean myo-inositol-1-phosphate (GmMIPS1) gene have also shown off-target effects, such as impaired development of seeds [61]. A study conducted by the product owner assessed the NK603 event as ‘substantially equivalent’ to its isogenic counterpart by a nutrient composition analysis (e.g. amino acids, fatty acids, vitamins and minerals) [62]. On the other hand, when ‘omics’ techniques were used to profile the proteome and metabolome of the same event, alterations in energy metabolism and oxidative stress pathways were found [53].

miRNA profiling methodologies are still evolving and responding to new information and hence, no consensus framework or pipelines have been established. We have chosen to use a more comprehensive analysis applying RNA-Seq as high-throughput unbiased method of miRNA target identification to uncover networks that require large-scale analysis instead of demonstrating individual miRNA:mRNA interactions (i.e. by RT-qPCR) because it misses the capacity for miRNAs to regulate complex gene networks [63]. We were able to confirm 77 out of 116 putative miRNA-predicted targets by this approach. Inconsistent correlation might be related to data observed amongst a pool of indirect changes in transcript abundance and, although it may assist in describing the predominant genes and pathways affected by a miRNA, it does not distinguish between direct targets [63]. It could also be related to the different target prediction algorithms, which are based around a model of interaction between the 5′-end of the miRNA called the ‘seed region’ and the 3′ untranslated region (3′-UTR) of the mRNA and this might not be the only interaction ruling between a miRNA sequence and its target. Indeed, increasing evidence demonstrates that targeting can also be mediated through sites other than the 3′-UTR and that seed region base pairing is not always required [63]. Nonetheless, identifying direct targets remains problematic given the potential modest effect on levels of some target transcripts and the fact that some miRNA targeting might occur predominantly at the level of translational repression [64].

Twenty novel miRNA sequences were found in the different maize samples. Two of them were observed in control samples only, one in RR sample only and another one in Bt samples alone. The other 15 novel miRNAs were found in all samples in similar abundance. The identification of novel miRNAs is a common approach in many plant miRNome profiling studies due to the yet relatively small-annotated miRNA database for plants [65]. Novel miRNA confirmatory analyses revealed amplifications for 7 miRNA primers but no differential profiles were observed. Target prediction of novel miRNA is possible through the use of bioinformatics database search according to sequence homology with known genes. Our target prediction revealed approximately 420 gene transcripts that could be regulated by these miRNAs. Metabolic pathway enrichment analysis of the 15 novel miRNAs shared among all samples showed GO terms that were assigned to lipid metabolism. Although several bioinformatics tools and algorithms have been developed to identify novel miRNAs in silico, there is still a need for detailed in vivo analyses to confirm their existence and expression. This is also the case for transgene-derived 22–24nt RNA sequences. We have found several potential transgenic miRNA which could not be verified using in silico prediction tools. Sequence variation in *cis*-regulatory elements which regulate genome transcription by binding to transcription factors might lead to higher expression of the associated miRNA locus only in the GM varieties. Whether such variation exists and is caused by transgene itself or the transgene-insertion process or other unrelated mutation, it needs additional experiment.

In addition to the novel miRNA sequences and the potential transgenic miRNA sequences, we found isomiRs for 96 miRNAs in our dataset. Frequently overlooked, isomiRs are several length and/or sequence variants of the same annotated miRNA. These variants were originally dismissed as experimental artefacts [66]. Because they vary in RNA length and/or sequence, many isomiRs are capable of showing major effects on miRNA targeting efficiency, AGO incorporation, loading into the RISC complex and stability [66].

We have previously alerted that unanticipated off-target adverse effects can be difficult to detect and it is not yet possible to reliably predict them using bioinformatics techniques [14]. Therefore, we aimed at identifying all unintended dsRNA molecules in the GM product through a semi-targeted qualitative profiling of small RNA molecules using next-generation sequencing in a comparative assessment between the GM and conventional parent. This approach would fit perfectly into the first phase of the molecular characterization step of current GMO RA. Nonetheless, identified unintended changes in miRNA content should be further verified and tested according to the guidelines we have previously suggested for improvements in risk assessment of GM crops or products containing dsRNA [14].

In this study, we have used a profiling approach which was able to identify several unintended miRNA sequences in single and stacked GM maize. These unintended miRNA sequences arose from differential expression of conserved miRNA families with a significant impact on metabolic pathways, novel miRNA sequences not yet annotated in the maize database, miRNA variants of unknown function (isomiRs) and potential transgenic miRNA sequences. Due to technical limitations, some of these sequences could not be verified or tested in silico and/or in vitro and therefore, require further in vivo analysis. All of these newly identified sequences can be a source of potential off-target effects and should be further considered in risk assessment frameworks [14]. In addition, safety studies cannot only rely on bioinformatics to predict potential miRNA gene targets. Our study showed that many of the predicted targets had no annotation in the maize GenBank and thus testing would be needed to specifically assess potential adverse effects on animal and human health and the environment.

## Conclusions

In summary, our data revealed 13 miRNAs that may play important roles in transgenic maize plants through the dys-regulation of gene targets. These 13 miRNAs generally modulate unique gene targets but co-ordinately target a few common pathways, with partially overlapping key nodal molecules, highlighting the redundancy of miRNAs and gene targets that focuses on reprogramming critical pathways to facilitate transgene expression in maize cells. Thus, the complex gene networks regulated by these miRNAs highlighted the challenges in validating these results and also to guiding hypothesis-driven downstream analysis. Twenty novel miRNAs, miRNA variants (isomiRs), and potential transgenic miRNA sequences were also detected in our experiment.

We have generated data from a level of information that has never been considered before in risk assessment of genetically modified plants reaching the market. Production of intended dsRNA molecules in GM crops is a legitimate concern due to their potential off-target effects on silencing genes other than those intended. We have provided an approach to properly assess the safety of dsRNA-producing GM organisms through the identification of all new intended and unintended dsRNA molecules in the GM product by profiling omics technique. Nonetheless, we have pointed to technical limitations and to areas in which further experimental validation is required. Experimental procedures will no doubt evolve as new information becomes available, but this should not be a reason not to apply our current scientific and technological knowledge to assessing the safe use of dsRNA in a commercial scale.

## Methods

### Plant material and growth chamber conditions

We have chosen commercial transgenic maize varieties tolerant to the herbicide glyphosate (“Roundup Ready”, RR) and Bt-expressing plants that have been extensively cultivated in Brazil and other southern hemisphere countries like Argentina and South Africa over the last years. Four maize varieties were used in this study. All are commonly found in the Brazilian market: AG8025RR2 (unique identifier MON-ØØ6Ø3-6 from Monsanto Company, glyphosate herbicide tolerance, Sementes Agroceres), AG8025PRO (unique identifier MON-89Ø34-3 from Monsanto Company, resistance to lepidopteran species, Sementes Agroceres) and AG8025PRO2 (unique identifier MON-89Ø34-3 × MON-ØØ6Ø3-6 from Monsanto Company, stacked event resistant to lepidopteran species and glyphosate-based herbicides, Sementes Agroceres). As a control, we have used the non-GM near-isogenic variety AG8025 (Sementes Agroceres). AG8025 seeds are hybrid progeny of the single cross between maternal endogamous lines “A” with the paternal endogamous line “B”. In this case, all hybrid progeny is 100% AB genotype. The stacked variety MON-89Ø34-3 × MON-ØØ6Ø3-6 expresses two insecticidal proteins (CRY1A.105 and CRY2AB2 proteins derived from *Bacillus thuringiensis* (Bt), which are active against certain lepidopteran insect species) and two identical EPSPS proteins, under the control of distinct regulatory sequences, providing tolerance to glyphosate-based herbicides. More information on the generation of these commercial transgenic varieties and their expression cassettes can be freely obtained at Biosafety Clearing House hosted by the UN Convention on Biological Diversity (http://bch.cbd.int/), searching by the variety unique identifiers provided above.

Seedlings were grown side by side in growth chambers (EletrolabTM model 202/3) set to 16-h light period and 25 °C (± 2 °C), using Plantmax HT substrate (Buschle & Lepper S.A.) and watered daily. No pesticide or fertilizer was applied. Around 50 plants were grown in climate chambers out of which 30 plants were randomly sampled per maize variety. The collected samples were separated into three groups of ten plants. The ten plants of each group were pooled and were considered one biological replicate. Maize leaves were collected at V4 stage (20 days after seedling). Leaf pieces were cut out, weighed and placed in 3.8-ml cryogenic tubes before immersion in liquid nitrogen. The samples were kept at − 80 °C until RNA isolation.

### miRNA isolation and deep sequencing

Total RNA was extracted from approximately 100 mg of frozen leaf tissue using the miRNeasy Mini Kit (Qiagen, Hilden, Germany) according to the manufacturer’s instructions. The isolated RNA was quantified using NanoDrop 1000 (Thermo Fisher Scientific, Wilmington, USA) and resolved in 1% MOPS denaturing gel. RNA samples (1 μg) were sent to FASTERIS SA (Geneva, Switzerland) for library construction and sequencing. Twelve small RNA (sRNA) libraries were constructed. Before library construction, samples were quantified with Qubit^®^ Fluorometric Quantitation (Life Technologies, California, USA) and quality control was accessed with Agilent 2100 Bioanalyzer (Agilent Technologies, California, USA). Briefly, the construction of sRNA libraries consisted of the following successive steps: (i) acrylamide gel purification of RNA fragments corresponding to sRNAs sizes (18–30 nt); (ii) ligation of the 3p and 5p adapters and indexes to the RNA in two separate subsequent steps, each followed by acrylamide gel purification; (iii) synthesis of cDNA followed by another acrylamide gel purification; and (vi) final step of PCR amplification to generate a cDNA colony template library for Illumina sequencing. The libraries sequencing was conducted using the TruSeq SBS Kit v3-HS (Illumina^®^) in an Illumina HiSeq 2500, with number of cycles of 1 × 50 + 7 (*single*-*end*) in one lane of the HiSeq Flow Cell v3 (Illumina^®^). Base-calling was performed using the pipelines HiSeq Control Software 2.2.38, RTA 1.18.61.0, CASAVA-1.8.2.

### Library analysis of small RNAs

All low-quality reads with FASTq values below 13 were removed, and 5′ and 3′ adapters, as well as index sequences, were trimmed using the Genome Analyzer Pipeline (Illumina) at Fasteris SA. In addition, quality control was performed with FastQC tool (http://www.bioinformatics.babraham.ac.uk/projects/fastqc/) and no bases with Phred quality score below 30 were found; thus no additional trimming was necessary. Reads outside the 18–26 nt range were excluded from the analysis. sRNAs belonging to rRNAs, tRNAs, snRNAs and snoRNAs, as well as chloroplastidial and mitochondrial sequences, derived from *Zea mays* and deposited in the Ensembl Plants and NCBI GenBank databases, were identified through mapping using the Bowtie2 v.2.2.4 software [67] and removed from the dataset.

### Identification of conserved and novel miRNAs

In order to determine conserved maize miRNA, the filtered sRNA sequences were mapped to the *Zea mays* genome (B73, RefGen_v3, release 25) deposited in the Ensembl Plants database using the Bowtie2 v.2.2.4 software [67], in which the complete alignment of the sequences was required, and no mismatches were allowed. In addition, reads with multiple alignments on the genome were further excluded from the analysis. The prediction of novel miRNA was performed using the miR-PREFeR pipeline [68] with the following parameters: (1) Maximum length of 250 nt for a miRNA precursor; (2) Reads depth cut-off of 200; (3) Maximum gap length of 50 nt between two *contigs* to form a candidate region; (4) Minimum and maximum length of the mature sequence of 21 and 24 nt, respectively; (5) No requirement of the star sequence to be expressed; and (6) Allow the mature star duplex to have only 2 nt 3′ overhangs. All annotated mRNA and miRNA in the B73, RefGen_v3, release 25 were excluded from the analysis. In addition, each putative novel miRNA was searched against the maize miRNAs deposited at miRbase (http://www.mirbase.org/). Moreover, the web-based tool MiPred [69] was used to classify the predicted novel miRNA as real, pseudo or not a miRNA precursor. To be considered a real precursor, the miRNA sequence has to have a Minimum Fold Energy (MFE) < − 20 kcal/mol and a *p*-value < 0.05. As for the pseudo precursor, at least one of the conditions has to be true.

The miR-PREFeR pipeline was also used to analyse possible miRNA variant sequences for the conserved miRNAs. The following parameters were used: (1) Maximum length of 300 nt for a miRNA precursor; (2) Reads depth cut-off of 2; (3) Maximum gap length of 100 nt between two *contigs* to form a candidate region; (4) Minimum and maximum length of the mature sequence of 18 and 24 nt, respectively; (5) No requirement of the star sequence to be expressed; and (6) Allow the mature star duplex to have only 2 nt 3′ overhangs. An annotation file containing 156 conserved maize miRNAs was provided in order to analyse only previously annotated miRNAs.

### Differential expression of miRNA

Read counts were retrieved using SeqMonk v0.29.0 (http://www.bioinformatics.babraham.ac.uk), in which probes were designed around known miRNA sequences in the reference genome. Only the reads that exactly overlapped the probes were considered for the read counting. The statistical analyses were performed through R language and environment [70] using the DESeq2 R package v1.8.1 [71] to normalize the read counts and perform the differential expression analysis. DESeq2 uses the Wald test for pairwise comparisons, as well as Benjamini–Hochberg method [72] for the *p*-value adjustment for multiplicity of testing.

### Validation of miRNAs by RT-qPCR

Validation of miRNA sequencing was performed by RT-qPCR amplification using designed primers targeting all differentially expressed miRNAs and novel miRNAs found in this study (Additional file 3). Poly(A) Tailing and cDNA synthesis was performed using the NCode™ VILO™ miRNA cDNA Synthesis Kit (Invitrogen, California, USA) according to the manufacturer’s recommendations. Forward primers consisted of the entire sequence for each of the mature miRNAs, while only one universal reverse primer, provided with the commercial kit, was used. The universal reverse primer annealed in the Poly(A) tail of the cDNA, ensuring that only Poly(A)-cDNA was quantified. Endogenous reference genes were based on previous work on validation [73]. The primers used for the endogenous genes are the same ones used in our previous work, but only the forward primer. The amplification efficiency was obtained from relative standard curves provided for each primer and calculated according to Pfaffl equations [74]. For each of the biological replicates, three independent cDNA syntheses were performed. The RT-qPCRs were performed using the EXPRESS SYBR^®^ GreenER™ miRNA qRT-PCR kit (Invitrogen) according to the manufacturer’s recommendations. The normalized relative quantity (NRQ) was calculated according to the Pfaffl equations [74]. Information on real-time data for this study has followed guidelines from the Minimum Information for Publication of Quantitative Real-Time PCR Experiments [75].

### Relative quantification of transgene transcripts

Quantification of cry1A.105, cry2Ab2 and espsps transcripts were performed on the same samples used for miRNA and mRNA sequencing. Reverse-transcription quantitative PCR (RT-qPCR) assay was adapted from previously developed assays for the specific detection of MON-89Ø34-3 × MON-ØØ6Ø3-6 transgenes [76] to hydrolysis ZEN–Iowa Black^®^ Fluorescent Quencher (ZEN/IBFQ) probe chemistry (Integrated DNA Technologies, INC Iowa, USA). cDNA was synthesized and amplification of each target gene was performed using the QuantiTect Probe RT-PCR Kit (Qiagen) according to the manufacturer’s instructions. The methodology (e.g. PCR conditions, primer and probe sequences, statistical analysis) was the same as described for our previous work [40].

### Prediction of miRNA targets and pathway enrichment analysis

The prediction of gene targets for the differentially expressed miRNAs was performed using the psRNAtarget online tool [77]. The following parameters were applied: (1) Maximum expectation: 3.0; (2) Length for complementarity scoring (hspsize): 20; (3) Target accessibility—allowed maximum energy to unpair the target site (UPE): 25; (4) Flanking length around target site for target accessibility analysis: 17 bp upstream and 13 bp downstream; and (5) Range of central mismatch leading to translational inhibition: 9–11 nt.

The miRNA target genes previously predicted were submitted to Single Enrichment Analysis (SEA) using the online tool agriGO v1.2 [78], with the following parameters: (1) Selected species: *Zea mays* ssp V5a; (2) Statistical test method: Hypergeometric; (3) Multi-test adjustment method: Hochberg (FDR); (4) Significance level of 0.05; (5) Minimum number of 5 mapping entries; and (6) Gene ontology type: Plant GO Slim. Following, the online tool REVIGO [79] was used to remove the redundant Gene Ontology (GO) terms. Only significant GO terms (False Discovery Rate (FDR) values < 0.05) were used, with the following parameters: (1) Allowed similarity: medium (0.7); (2) Database with GO term sizes: *Zea mays*; e (3) Semantic similarity measure: SimRel.

### Confirmatory analysis for miRNA-predicted target relation

Total RNA was also isolated from the same samples using the RNeasy Plant Mini Kit (Qiagen, Hilden, Germany) according to the manufacturer’s instructions and submitted to transcriptome sequencing at FASTERIS SA (Geneva, Switzerland). Sequencing of the cDNA libraries was conducted using the HiSeq SBS Kit v4 (Illumina^®^) in an Illumina HiSeq 2500, with number of cycles of 2 × 125 + 7 (paired-end) in one lane of the HiSeq Flow Cell v4 (Illumina^®^). Base-calling was performed using the pipelines HiSeq Control Software 2.2.38, RTA 1.18.61.0, CASAVA-1.8.2.

All low-quality reads with FASTq values below 13 were removed, and 5′ and 3′ adapter, as well as index sequences, were trimmed using the Genome Analyzer Pipeline (Illumina) at Fasteris SA. Moreover, quality control with FastQC tool (http://www.bioinformatics.babraham.ac.uk/projects/fastqc/) was performed and no bases with Phred quality score below 30 were found; thus no additional trimming was necessary.

The filtered reads were mapped against the *Zea mays* genome (B73, RefGen_v3, release 25), deposited in the Ensembl plants database [80], using the TopHat2 v2.1.0 tool [81], with a maximum of two mismatches, in which gaps count as mismatches. Pairwise differential analyses were conducted using the Tuxedo package [82], which uses Cufflinks v.2.2.0, Cuffmerge and Cuffdiff tools, and is able to assemble transcripts, estimate their abundance, and test the expression and differential regulation of RNA-Seq libraries.

## Additional files

**Additional file 1.** Read size distribution of all sequenced sRNA libraries.

**Additional file 2.** Normalized read count of the sRNA libraries mapped to conserved *Zea mays* miRNAs.

**Additional file 3.** List of the primers used to amplify the differentially expressed conserved miRNAs and the putative novel miRNAs.

**Additional file 4.** Output of the psRNAtarget for the prediction of conserved miRNA targets.

**Additional file 5.** Expression values of the conserved miRNA targets obtained by RNA-Seq data.

**Additional file 6.** List of the predicted novel miRNAs.

**Additional file 7.** Predicted structure of all 20 novel miRNAs using miR-PREFeR pipeline.

**Additional file 8.** Output of the psRNAtarget for the prediction of novel miRNA targets.

**Additional file 9.** Results of the enrichment analysis of the miRNA targets from the 15 novel miRNAs found in all four varieties.

## Abbreviations

Bt: *Bacillus thuringiensis*
cDNA: complementary DNA
DNA: deoxyribonucleic acid
dsRNA: double-stranded RNA
EFSA: European Food Safety Authority
FC: fold change
GM: genetically modified
GMO: genetically modified organism
GO: gene ontology
miRNA: micro RNA
PCR: polymerase chain reaction
RISC: RNA-induced silencing complex
RNA: ribonucleic acid
RNAi: RNA interference
RR: roundup ready
rRNA: ribosomal RNA
RT-qPCR: quantitative reverse transcription PCR
siRNA: small interfering RNA
snoRNA: small nucleolar RNAs
snRNA: small nuclear RNAs
tRNA: transfer RNA
