# Supplementary material for: Systematic miRNome profiling reveals differential microRNAs in transgenic maize metabolism
Source: Environ Sci Eur. 2018 Sep 19;30(1):37. doi: 10.1186/s12302-018-0168-7 (PMC6153861; doi:10.1186/s12302-018-0168-7)

zma-miRX01

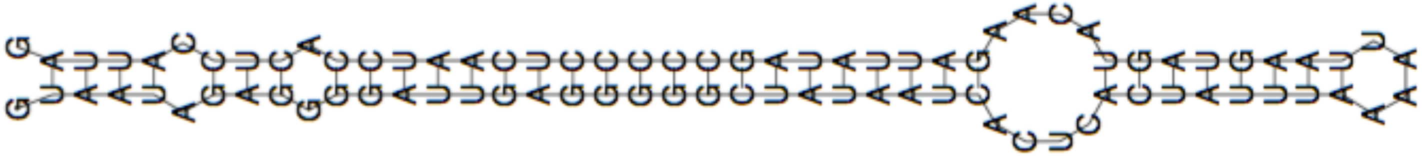

zma-miRX02

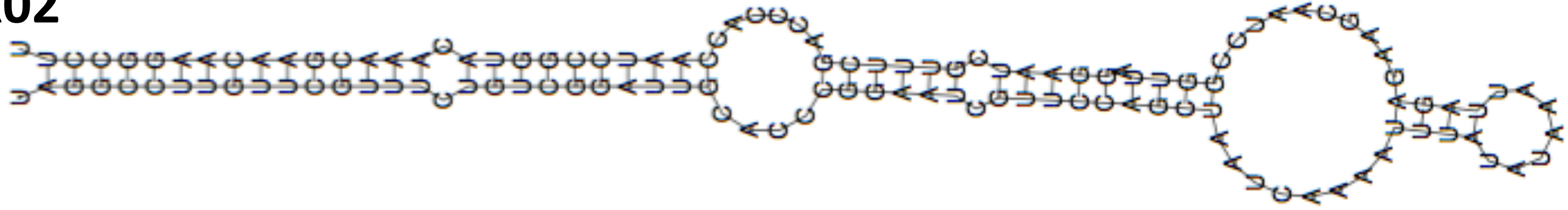

zma-miRX03

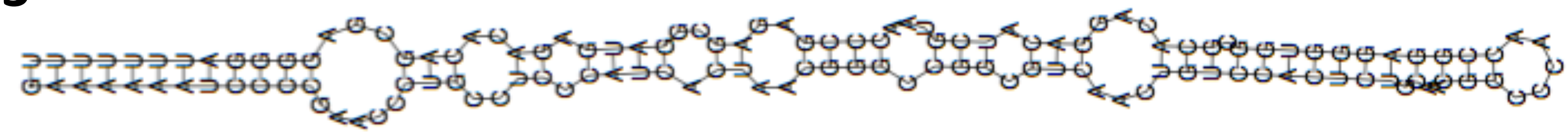

zma-miRX04

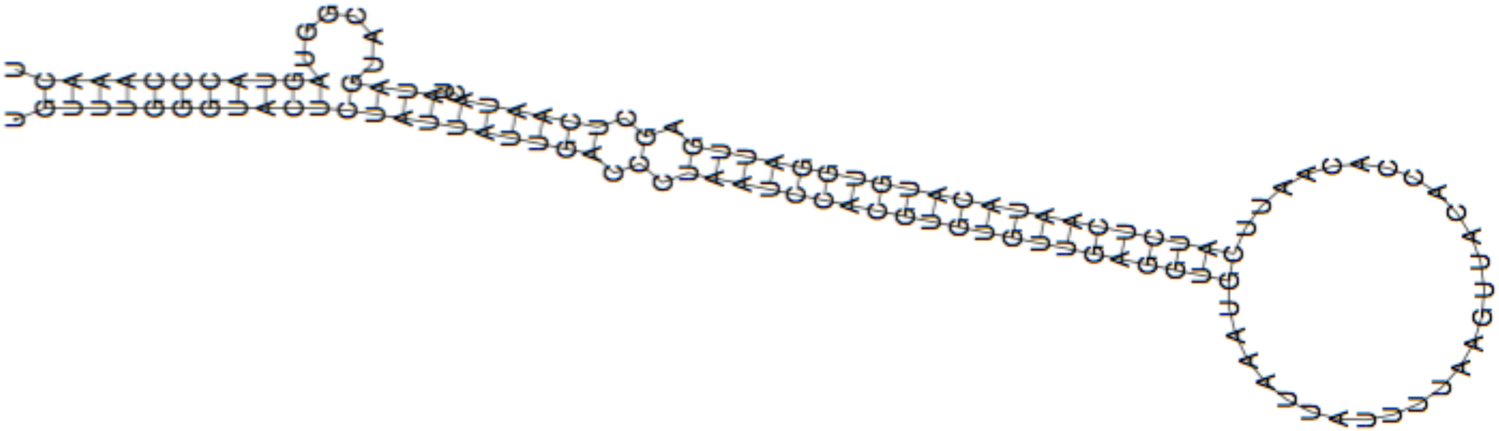

zma-miRX05

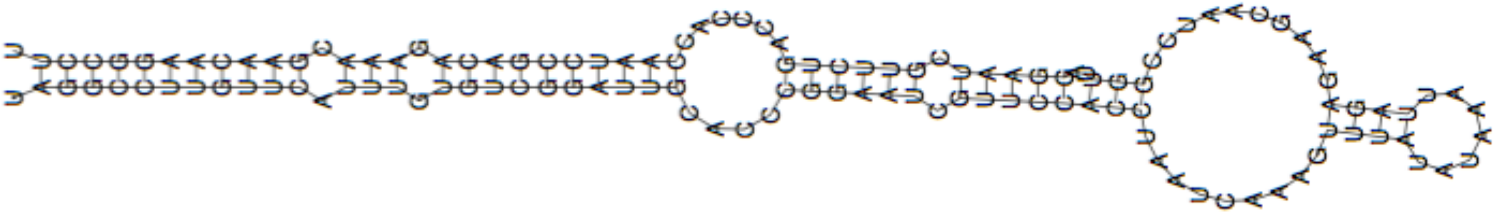

zma-miRX06

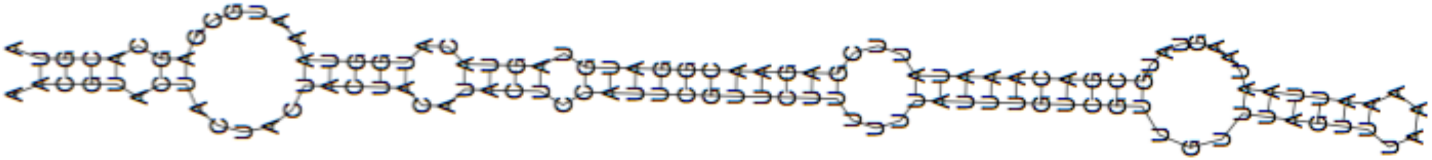

zma-miRX07

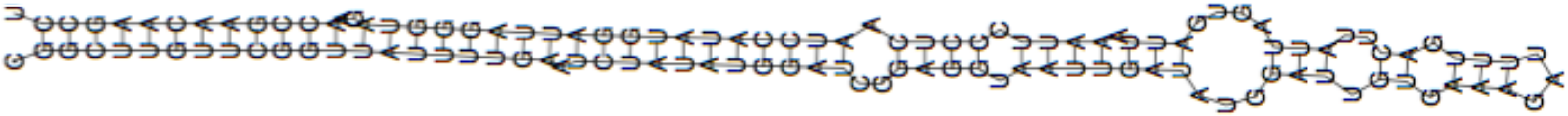

zma-miRX08

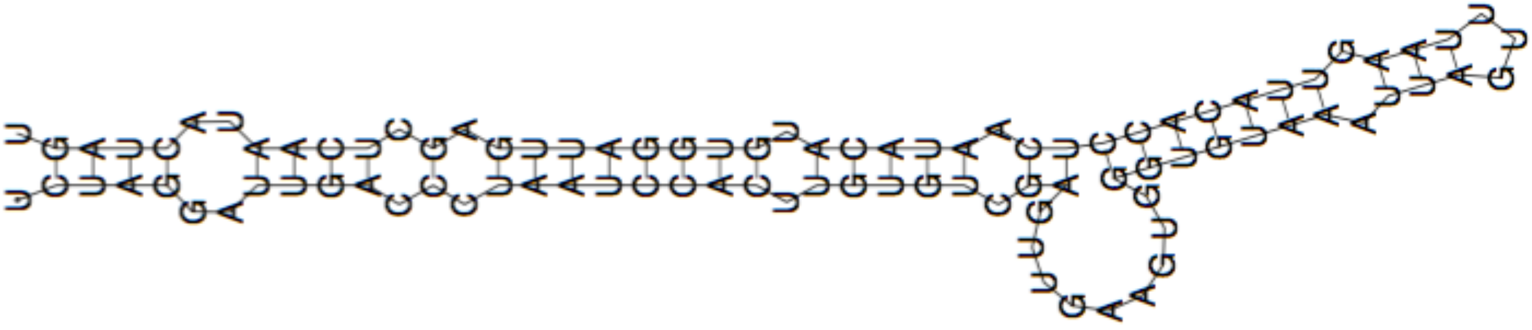

zma-miRX09

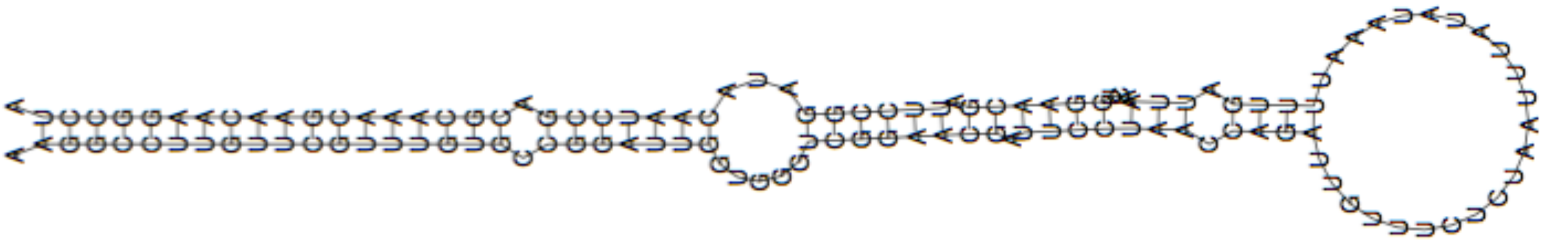

zma-miRX10

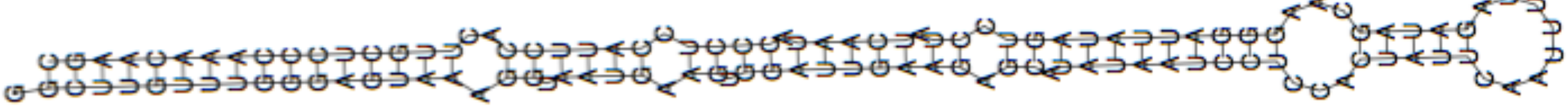

zma-miRX11

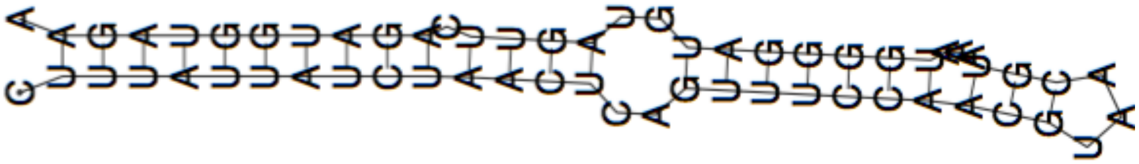

zma-miRX12

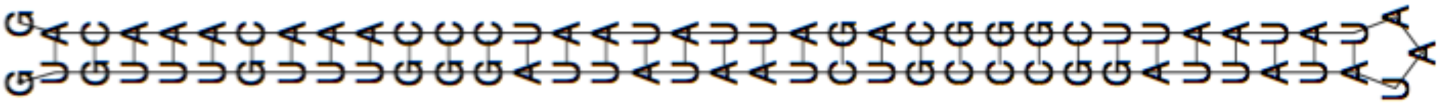

zma-miRX13

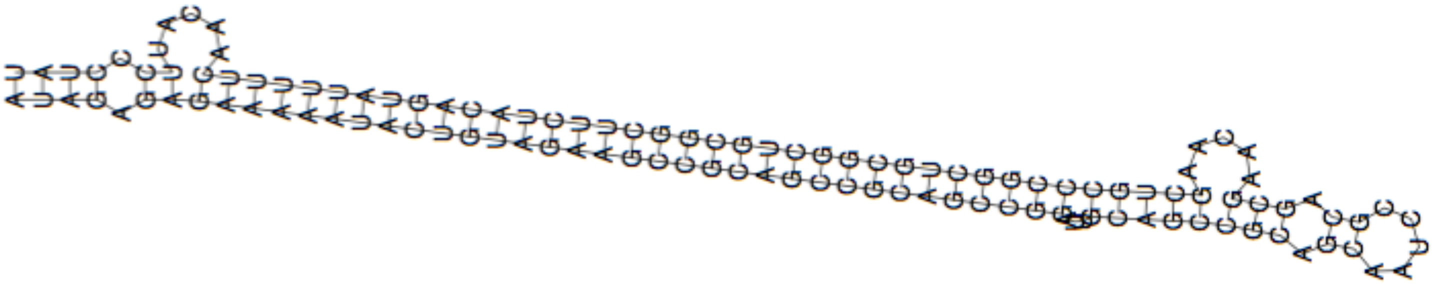

zma-miRX14

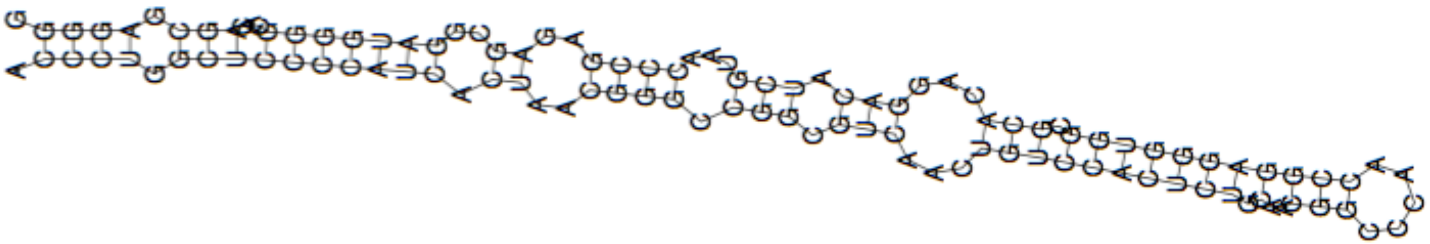

zma-miRX15

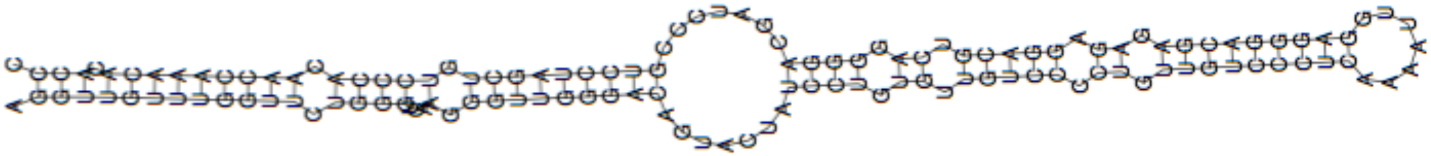

zma-miRX16

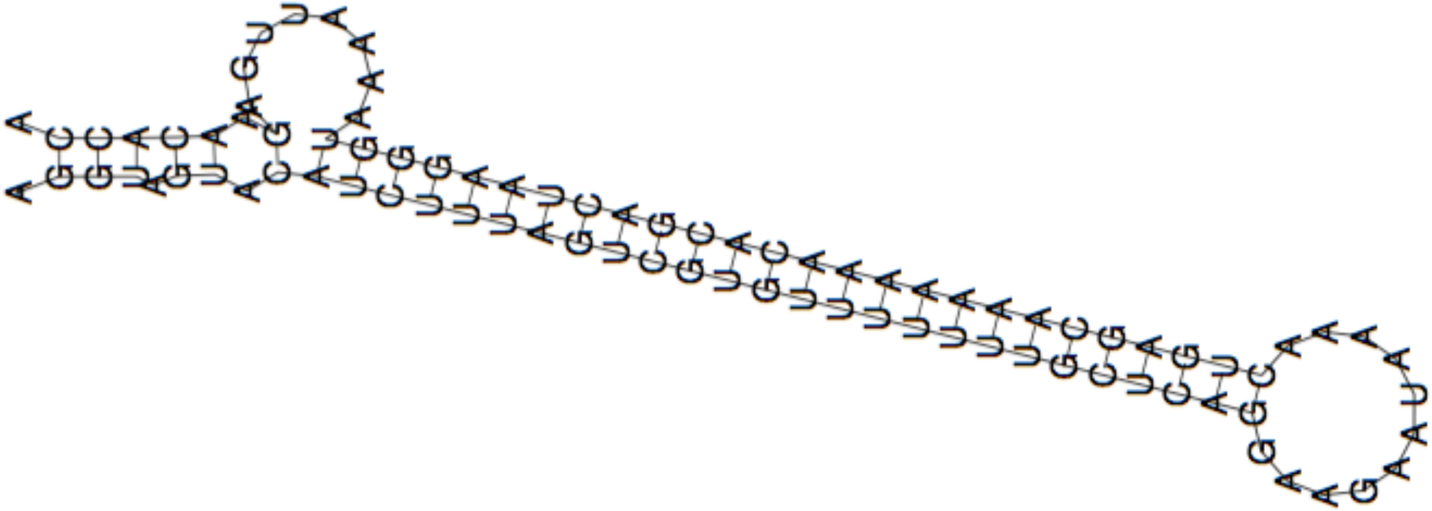

zma-miRX17

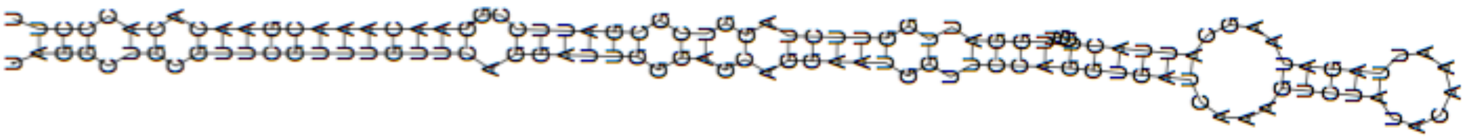

zma-miRX18

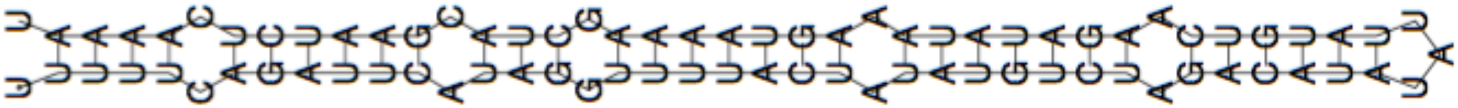

zma-miRX19

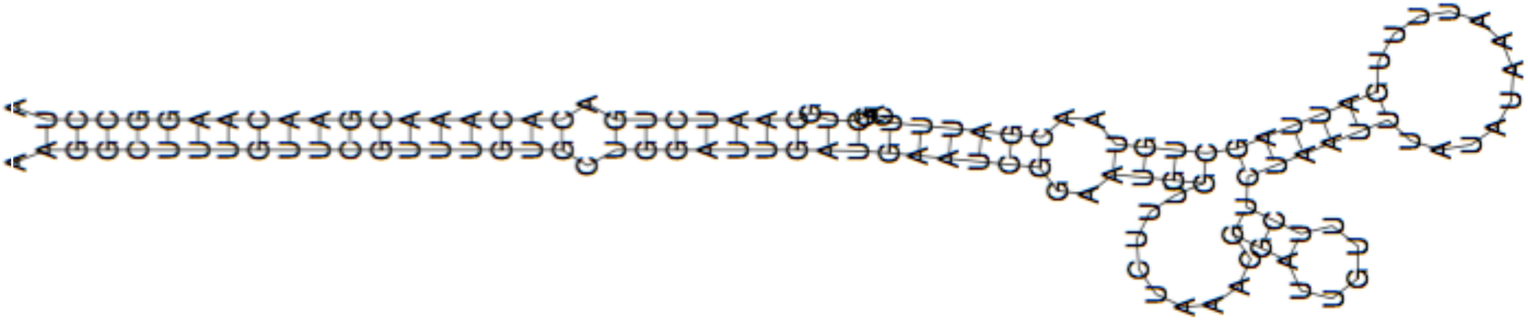

zma-miRX20

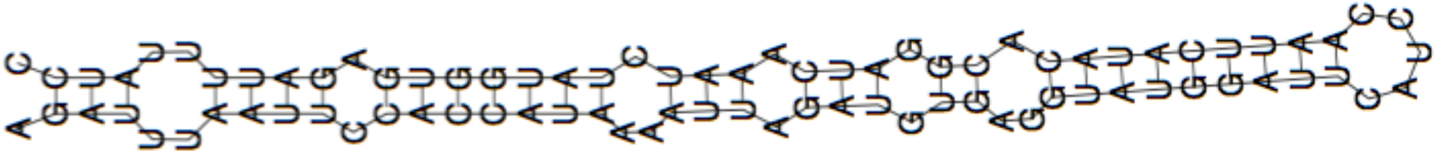

Supplement: Supplementary file 7 — Additional file 7. Predicted structure of all 20 novel miRNAs using miR-PREFeR pipeline. [file 12302_2018_168_MOESM7_ESM.pdf]
